# Variational Autoencoders for Medical Image Segmentation: A Review

**DOI:** 10.3390/jimaging12100481

**Published:** 2026-10-03

**Authors:** Nabil Marzoug, Moulay A. Akhloufi

**Affiliations:** Perception, Robotics, and Intelligent Machines (PRIME), Department of Computer Science, Université de Moncton, Moncton, NB E1A 3E9, Canada; moulay.akhloufi@umoncton.ca

**Keywords:** variational autoencoders, medical image segmentation, synthetic data generation, data augmentation, latent representations, deep learning

## Abstract

Medical image segmentation plays an important role in healthcare because it transforms raw scans into useful information for diagnosis and treatment planning. Despite the strong performance of deep learning, most supervised segmentation methods require large amounts of pixel-level annotations and may become less reliable when imaging modalities are incomplete or when prediction uncertainty is not properly considered. Variational autoencoders (VAEs) are deep learning models that were proposed to address some of these limitations, as they can learn probabilistic latent representations capable of capturing meaningful structure in medical images. This review provides a structured survey of how VAEs are applied to medical image segmentation and organizes them around five roles: generating synthetic samples for data augmentation, integrating variational representations into segmentation networks for representation learning and regularization, localizing pathology through reconstruction-based anomaly detection, fusing multiple modalities in a shared latent space for missing-modality segmentation, and learning anatomical shape priors for segmentation correction and quality control. In this work, the reviewed literature includes both approaches where the VAE directly contributes to the segmentation and related applications that support the segmentation pipeline, such as data generation, preprocessing or weak localization. We also summarize the main datasets and evaluation metrics used in the field and discuss current challenges, including blurry reconstructions, the use of hybrid architectures in several reviewed studies, the strong concentration of studies on brain MRI, the limited use of uncertainty, and the possible complementary roles of VAEs alongside diffusion and foundation models. Overall, the studies reviewed here suggest that VAEs can serve as parts of larger systems, where they provide structured representations and help handle missing modalities. Conditional models trained to predict masks can also generate multiple plausible segmentations, which may reflect variation among annotations when the training data capture it.

## 1. Introduction

Medical image segmentation is an area in which the aim is to identify the boundaries of organs, tissues, and lesions in medical scans. It is an essential step in image analysis because these regions can then be used to obtain quantitative information for diagnosis, treatment planning, and patient follow-up. Over the last decade, deep learning has become the main approach for this task, with architectures such as U-Net [1] and its many variants achieving strong results across different imaging modalities. Despite this progress, most of these models depend heavily on supervised learning. They require large datasets with pixel-level annotations, which are difficult and expensive to obtain because they must usually be prepared by medical experts. Their performance can also decrease when the test images differ from the training data, when some imaging modalities are unavailable, or when information about prediction uncertainty is required.

Generative models provide several ways to address these limitations. Generative adversarial networks (GANs) [2], variational autoencoders (VAEs) [3], and more recently diffusion models have been used to increase limited datasets, learn representations from unlabeled images, and model the distribution of normal anatomy. VAEs have several properties that make them particularly useful for segmentation. Instead of mapping an image to a single latent representation, a VAE represents it as a probability distribution in a lower dimensional space and reconstructs the image from samples drawn from this distribution. This provides a structured and continuous latent space that can be regularized, a probabilistic representation that can model variability in the data, and relatively stable training compared with adversarial models. Because of these properties, VAEs can contribute to segmentation in several ways beyond simply generating additional training images.

From the literature reviewed in this survey, five main roles of the VAE can be identified. The first is generative augmentation, where the VAE generates synthetic images or paired image and mask samples to augment a limited training set. The second is latent representation integration and regularization, where the variational representation is incorporated into a segmentation framework to support representation learning and mask prediction. A third role is reconstruction-based anomaly localization, where the VAE is usually trained to model normal anatomy and abnormal regions are identified from reconstruction error or related deviations. The fourth role is multimodal latent fusion and missing-modality segmentation, where information from several modalities is combined in a shared latent space to allow segmentation when some expected inputs are missing. The fifth role is anatomical shape priors and segmentation quality control, where the VAE learns the distribution of plausible anatomical masks and is used to detect or correct unrealistic segmentation outputs. These five roles form the main taxonomy used throughout this survey. They appear across different clinical applications, from brain and lung imaging to retinal and multimodal imaging, showing that the function of the VAE often depends more on the available data and the objective of the model than on the anatomy itself.

Many previous works from the literature have addressed topics that partially overlap with the scope of the present work, but they differ in terms of the generative model, the medical imaging task, or the way the literature is organized. Yi et al. [4] reviewed the use of GANs across several medical imaging applications, including image synthesis, reconstruction, segmentation and data augmentation. More specifically for segmentation, Iqbal et al. [5] provided a review of GAN-based approaches for biomedical image segmentation. Similarly, Kazerouni et al. [6] reviewed diffusion models across different medical imaging applications, while Yaseen et al. [7] focused more specifically on diffusion-based medical image segmentation. Although these reviews cover segmentation, their main focus is on GANs or diffusion models rather than on the role of VAEs inside the segmentation pipeline.

Other reviews are closer to the scope of this work. Kebaili et al. [8] reviewed VAEs, GANs and diffusion models for medical image augmentation, where segmentation is mainly considered as a downstream task benefiting from generated data. Zhou et al. [9] focused specifically on brain tumor segmentation with missing MRI modalities and reviewed different families of approaches for handling incomplete inputs, without focusing specifically on VAEs. More recently, Yeoh et al. [10] reviewed the trends and applications of VAEs in medical imaging across several tasks, including image synthesis, classification, anomaly detection, registration and segmentation. Yeoh et al. [11] further organized VAE applications according to anatomical regions and considered several downstream tasks such as segmentation, synthesis, classification and anomaly detection. In these VAE reviews, segmentation therefore remains one application among several.

In contrast, the present review focuses specifically on the different ways in which VAEs contribute to medical image segmentation. Instead of organizing the literature mainly according to the generative model, anatomical region or general medical imaging task, we organize the reviewed studies according to the role played by the VAE in the segmentation pipeline. Particular attention is also given to multimodal segmentation and missing-modality settings, where the shared latent representation of VAEs can be directly used for segmentation. Table 1 summarizes the main differences between the previous reviews and the present work.

This survey aims to address this gap. Its main contributions are the following:

- We provide a focused review of variational autoencoders in medical image segmentation, that covers studies published between 2018 and 2026.
- We introduce a taxonomy based on five main roles of the VAE: generative augmentation, latent representation integration and regularization, reconstruction-based anomaly localization, multimodal latent fusion and missing-modality segmentation, and anatomical shape priors and segmentation quality control. This taxonomy is used to organize and compare the reviewed methods.
- We examine the literature across different imaging areas, including radiography, computed tomography, magnetic resonance imaging, and retinal imaging, and summarize the main datasets, architectures, and reported results in each area.

### Review Methodology and Search Strategy

This article is a structured narrative review of variational autoencoders (VAEs) in medical image segmentation. It aims to compare representative methods and organize them according to the five roles defined in this article, rather than to provide an exhaustive systematic review or meta-analysis. Literature searching began in June 2025. The final documented searches of PubMed, IEEE Xplore, and Scopus were conducted on 25 September 2026, with publication years limited to 2018–2026. Earlier foundational studies were retained as background references when relevant.

The database queries combined three concepts: variational autoencoders, segmentation, and medical imaging. Database-specific syntax was used for the same set of concepts. Table 2 reports the search date, publication-year limit, and number of records exported from each database. The database-specific queries are reproduced below the table. Google Scholar was used separately for supplementary targeted searching and citation tracking.

**PubMed query.**

("variational autoencoder"[Title/Abstract] OR

"variational auto-encoder"[Title/Abstract] OR

VAE[Title/Abstract] OR

"conditional variational autoencoder"[Title/Abstract])

AND (segment*[Title/Abstract] OR

"medical image segmentation"[Title/Abstract] OR

"lesion segmentation"[Title/Abstract] OR

"organ segmentation"[Title/Abstract])

AND (medical[Title/Abstract] OR MRI[Title/Abstract] OR

"magnetic resonance"[Title/Abstract] OR CT[Title/Abstract] OR

"computed tomography"[Title/Abstract] OR

radiograph*[Title/Abstract] OR "X-ray"[Title/Abstract] OR

ultrasound[Title/Abstract] OR retinal[Title/Abstract] OR

fundus[Title/Abstract] OR OCTA[Title/Abstract])

**IEEE Xplore query.**

("variational autoencoder" OR "variational auto-encoder" OR VAE OR

"conditional variational autoencoder")

AND (segment* OR "medical image segmentation" OR

"lesion segmentation" OR "organ segmentation")

AND (medical OR MRI OR "magnetic resonance" OR CT OR

"computed tomography" OR radiograph* OR "X-ray" OR

ultrasound OR retinal OR fundus OR OCTA)

**Scopus query.**

TITLE-ABS-KEY(

("variational autoencoder" OR "variational auto-encoder" OR VAE OR

"conditional variational autoencoder")

AND (segment* OR "medical image segmentation" OR

"lesion segmentation" OR "organ segmentation")

AND (medical OR MRI OR "magnetic resonance" OR CT OR

"computed tomography" OR radiograph* OR "X-ray" OR

ultrasound OR retinal OR fundus OR OCTA) )

The searches exported 837 records: 80 from PubMed, 387 from IEEE Xplore, and 370 from Scopus. Cross-database duplicates were identified by DOI and then by title after lowercasing and removing accents, punctuation, and spaces. A discrepant DOI associated with an identical title was checked individually. The 150 duplicate entries comprised 65 PubMed–Scopus pairs, 59 IEEE Xplore–Scopus pairs, and 13 records indexed by all three databases, accounting for 26 duplicate entries. This left 687 unique database records. Distinct Scopus records sharing a generic proceedings-volume title were retained rather than merged by title alone.

The titles and exported abstracts of all 687 unique records were screened against the criteria described below. At this stage, 481 records were excluded, including proceedings front matter and reviews; records outside medical imaging; and papers whose proposed method did not show a relevant variational-model contribution to segmentation or a closely related application. The remaining 206 records were retained as candidates for further assessment and possible inclusion in the narrative synthesis. A paper was not excluded merely because its title omitted “VAE”: descriptions of probabilistic segmentation models and mask-generating latent models were also considered. Decisions and exclusion reasons were recorded for each database record.

We considered primary studies dated 2018–2026, prioritizing peer-reviewed journal and conference papers. For borderline methods, the model architecture and training objective were examined rather than relying only on the presence of “VAE” in the title or abstract. A study qualified for a core role when it evaluated a VAE or an explicitly VAE-derived latent-variable model in a segmentation-related task. Relevant outcomes included predicted masks; the effect of a VAE component on a segmentation network; downstream segmentation after synthetic augmentation or missing-modality completion; lesion-mask evaluation of reconstruction-based anomaly localization; and anatomical shape priors or quality-control mechanisms applied to segmentation masks. A deterministic or adversarial autoencoder was not treated as a VAE solely because it had an encoder–decoder architecture or a regularized latent space. When a study evaluated both a VAE and another type of autoencoder, their results were distinguished. Studies of synthesis or weak localization without direct segmentation evaluation were discussed separately as related applications, without attributing a segmentation improvement to them. Borderline classifications and role assignments were discussed between the authors.

For studies considered for detailed discussion, full texts were used to assess the actual role of the variational component and the outcomes evaluated. The 206 records retained after title and abstract screening therefore represent a candidate pool for further assessment rather than a set of studies that all underwent formal systematic full-text eligibility assessment. Review articles, dataset descriptions, and foundational methodological papers were used as background sources but were not counted as primary studies.

Selection for the narrative synthesis was purposive. Priority was given to work that introduced a distinct design, provided a more complete evaluation of a previously described method, or represented a functional role or imaging domain covered in this review. Of the 49 primary studies discussed, 30 were present in the three database exports. The other 19 were identified through supplementary Google Scholar searching and were absent from those exports. The supplementary searches combined “variational autoencoder” or “VAE” with “medical image segmentation” and modality- or anatomy-specific terms, and citations of selected studies were also examined. These supplementary searches were iterative and purposive rather than systematic.

Forty-one studies are organized under the five core roles and eight are presented as related applications. As the selection was purposive for a narrative synthesis, studies not chosen for detailed discussion were not necessarily ineligible. Figure 1 shows the database and supplementary identification routes separately.

The final selection of studies and borderline taxonomy assignments for the narrative synthesis were discussed between the authors.

As illustrated in Figure 2, the remainder of this review is organized as follows. Section 2 introduces the main concepts behind autoencoders, variational autoencoders, and their multimodal extensions. Section 3 describes the five roles of the VAE in greater detail. Section 4, Section 5, Section 6, Section 7 and Section 8 review the literature across radiography, computed tomography, magnetic resonance imaging, retinal imaging, and multimodal applications. Section 9 summarizes the datasets and evaluation metrics used throughout the literature. The main limitations and future research directions are discussed in Section 10, before the review is concluded in Section 11.

## 2. Background: VAE Fundamentals and Variants

### 2.1. From Autoencoders to Variational Autoencoders

Autoencoders (AEs) are a type of neural network that were designed to learn compressed representations of the data through the encoder and the decoder architecture [12]. The encoder $f_{\phi }$ maps input data *x* to a lower-dimensional latent representation $z=f_{\phi }\left(x\right)$, while the decoder $g_{\theta }$ attempts to reconstruct the original input as $\tilde{x}=g_{\theta }\left(z\right)$. Training process of the autoencoder tries to minimize the reconstruction error:

(1) $$L_{AE}={∥x-g_{\theta }\left(f_{\phi }\left(x\right)\right)∥}^{2}$$

While autoencoders are considered to be very good at dimensionality reduction and feature learning, they suffer from a critical limitation for generative tasks. The latent space learned by autoencoders lacks structure, meaning that points in the latent space that were not seen during training may decode into unrealistic outputs. The latent representations are deterministic and can form disconnected clusters, which makes smooth interpolation between data points and the generation of new realistic samples less reliable. This limitation motivated the development of Variational Autoencoders.

### 2.2. VAE Architecture and Training

Variational autoencoders (VAEs), introduced by [3] and later comprehensively reviewed by [13], are among the most widely used deep generative models in machine learning. Unlike conventional autoencoders, which learn deterministic latent representations for data compression, VAEs adopt a probabilistic framework that enables both representation learning and data generation. Instead of mapping each input to a fixed latent vector, the encoder learns a probability distribution over the latent variables, allowing the model to represent variability in the latent space. This probabilistic formulation results in a continuous and structured latent space from which new samples can be generated by sampling latent vectors from a predefined prior distribution, typically a standard Gaussian.

A VAE consists of two neural network components that are trained jointly: an encoder and a decoder, as illustrated in Figure 3. The encoder maps an input sample *x* to an approximate posterior distribution over the latent variable *z*, denoted by $q_{\phi }\left(z|x\right)$. In practice, this distribution is usually modeled as a multivariate Gaussian parameterized by its mean μ and variance $\sigma^{2}$. Rather than producing a single latent representation, the encoder estimates these distribution parameters, allowing different latent vectors to be sampled from the same input. The encoder can therefore be expressed as

(2) $$q_{\phi }\left(z|x\right)=N\left(z;\mu_{\phi }\left(x\right),\sigma_{\phi }^{2}\left(x\right)\right).$$

The decoder performs the inverse mapping by reconstructing the original input from a latent sample drawn from the learned distribution. Specifically, it models the conditional likelihood $p_{\theta }\left(x|z\right)$ and learns to generate samples that closely resemble the original data [14]. As shown in Figure 3, the latent vector is obtained through the reparameterization trick, while the KL divergence regularizes the latent distribution toward the prior distribution. Together, these components enable the model to learn informative latent representations while preserving its generative capability.

The encoder and decoder are optimized by maximizing the Evidence Lower Bound (ELBO), which provides a tractable objective for learning the model parameters:

(3) $$L\left(\theta ,\phi ;x\right)=E*q*\phi (z|x)\left[\log p_{\theta }\left(x|z\right)\right]-D_{KL}\left(q_{\phi }\left(z|x\right)\|\;p\left(z\right)\right).$$

The ELBO consists of two complementary terms. The first term, referred to as the reconstruction term, measures how accurately the decoder can reconstruct the input from the sampled latent representation. Maximizing this term encourages the latent variables to retain the essential information required to reproduce the original data. The second term is the Kullback–Leibler (KL) divergence, which measures the discrepancy between the approximate posterior learned by the encoder and the prior distribution p(z), commonly chosen as N(0,I) [13]. Minimizing this divergence regularizes the latent space by encouraging the learned representations to follow a smooth and continuous distribution, thereby improving the model’s ability to generate realistic and diverse samples.

Since sampling from a probability distribution is not directly differentiable, VAEs employ the reparameterization trick to enable gradient-based optimization. The latent variable is expressed as

(4) $$z=\mu_{\phi }\left(x\right)+\sigma_{\phi }\left(x\right)⊙\epsilon ,\;\epsilon ∼N\left(0,I\right),$$

where ϵ is sampled from a standard Gaussian distribution. This formulation separates the stochastic sampling process from the learnable network parameters, allowing gradients to propagate through the encoder during backpropagation while preserving the probabilistic nature of the latent representation.

The combination of probabilistic latent representations, latent space regularization, and end-to-end optimization makes VAEs a powerful framework for generative modeling. After training, new samples can be synthesized by sampling latent vectors from the prior distribution and passing them through the decoder. Furthermore, the learned latent space often captures meaningful semantic characteristics of the data, enabling smooth interpolation between samples, controlled data generation, and effective representation learning. These properties have led to the widespread adoption of VAEs in applications such as medical image synthesis, data augmentation, anomaly detection, image reconstruction, molecular generation, and multimodal learning. Despite their advantages, VAEs may produce blurrier outputs than adversarial models such as GANs because the reconstruction objective tends to average over multiple plausible solutions. Nevertheless, their stable training procedure, principled probabilistic formulation, and well-structured latent space have established VAEs as one of the foundational architectures in modern generative artificial intelligence.

### 2.3. Variational AutoEncoder Variants

Several variants of the VAE have been proposed over the years to improve different aspects of the original model, including the quality of generated samples, the structure of the latent space and the integration of different modalities. In this subsection, we focus on the VAE variants that are most relevant to the medical image segmentation studies discussed in this review.

#### 2.3.1. Conditional Variational AutoEncoder (CVAE)

The Conditional VAE was introduced by Sohn et al. [15] to allow the generation process to be conditioned on additional information such as class labels. In standard VAE, the model learns to generate samples from the data distribution without any control over specific attributes. CVAE addresses this by adding a condition variable *c* into both the encoder and decoder. The encoder becomes $q_{\phi }\left(z|x,c\right)$ and the decoder becomes $p_{\theta }\left(x|z,c\right)$. The objective function is modified as:

(5) $$L_{CVAE}=E_{q_{\phi }\left(z|x,c\right)}\left[\log p_{\theta }\left(x|z,c\right)\right]-D_{KL}\left(q_{\phi }\left(z|x,c\right)\|\;p\left(z|c\right)\right)$$

This allows the model to generate samples with specific attributes by providing the desired condition during generation. For example, in image generation tasks, we can put the condition on class labels to generate images of a specific category.

#### 2.3.2. β-VAE

The β-VAE was proposed by Higgins et al. [16] to learn disentangled representations in the latent space. Disentanglement means that each dimension of the latent vector captures a separate and independent factor of variation in the data. The key modification is adding a hyperparameter β to control the weight of the KL divergence term:

(6) $$L_{\beta -VAE}=E_{q_{\phi }\left(z|x\right)}\left[\log p_{\theta }\left(x|z\right)\right]-\beta \cdot D_{KL}\left(q_{\phi }\left(z|x\right)\|\;p\left(z\right)\right)$$

When β>1, the model puts more pressure on the latent distribution to match the prior, which encourages the latent dimensions to become more independent. However, increasing β too much can hurt reconstruction quality because the model focuses more on matching the prior than on accurate reconstruction. Thus, finding the right balance is important for getting good disentanglement without losing too much reconstruction quality.

#### 2.3.3. Vector Quantized VAE (VQ-VAE)

The VQ-VAE introduced by van den Oord et al. [17] takes a different approach by using a discrete latent space instead of a continuous one. This discrete representation can also reduce some forms of posterior collapse that may occur in standard VAEs, where the decoder learns to ignore the latent code. In VQ-VAE, the encoder output $z_{e}\left(x\right)$ is mapped to the nearest vector in a learned codebook $e\in R^{K\times D}$, where *K* is the number of embedding vectors and *D* is the dimension of each vector. The quantization operation is:

(7) $$z_{q}\left(x\right)=e_{k},\;\mathrm{where}\;k=\arg\min_{j}{∥z_{e}\left(x\right)-e_{j}∥}_{2}$$

The loss function consists of three terms:

(8) $$L_{VQ-VAE}=\log p\left(x|z_{q}\left(x\right)\right)+∥sg\left[z_{e}\left(x\right)\right]{-e∥}_{2}^{2}+\beta {∥z_{e}\left(x\right)-sg\left[e\right]∥}_{2}^{2}$$

where sg[·] is the stop-gradient operator. The first term is the reconstruction loss, the second term moves the codebook vectors towards the encoder outputs, and the third term encourages the encoder to commit to codebook vectors. Since the quantization operation is not differentiable, gradients are passed through using a straight-through estimator. VQ-VAE-2 [18] extends this idea by using several levels of discrete latent representations at different scales. This allows the model to capture both local details and more global image information. However, since VQ-VAE-2 is not commonly used in the medical segmentation studies reviewed here, we do not discuss it further.

#### 2.3.4. VAE-GAN

Larsen et al. [19] proposed combining VAE with GAN to get the benefits of both models. The main idea is to replace the pixel-wise reconstruction loss in VAE with a feature-wise loss computed using a discriminator network. The VAE-GAN model has three components: an encoder, a decoder (which also acts as the generator), and a discriminator. The decoder is trained to not only reconstruct inputs but also to generate samples that can fool the discriminator. The loss function combines the VAE objective with the GAN objective:

(9) $$L_{VAE-GAN}=L_{prior}+L_{recon}^{feature}+L_{GAN}$$

where $L_{recon}^{feature}$ is computed as the difference between feature representations of the input and reconstruction in the discriminator. This combination helps produce sharper images compared to standard VAE while maintaining the benefits of a structured latent space.

#### 2.3.5. Hierarchical VAE (HVAE)

Hierarchical VAEs extend the standard VAE by using multiple layers of latent variables arranged in a hierarchy [20]. The generative model factorizes as:

(10) $$p_{\theta }\left(x,z_{1},\ldots ,z_{L}\right)=p_{\theta }\left(x|z_{1},\ldots ,z_{L}\right)\prod_{l=1}^{L}p_{\theta }\left(z_{l}|z_{l+1},\ldots ,z_{L}\right)$$

where $z_{L}$ is the top-level latent variable. The use of several latent levels allows hierarchical VAEs to capture information at different scales, including both local details and more global structures in the data. The Ladder VAE [21] follows a similar idea by combining bottom-up information from the encoder with top-down information from higher latent levels.

#### 2.3.6. Multimodal VAEs (MVAE, MMVAE, MoPoE)

The variants presented so far all operate on a single input modality. However, medical imaging is often multimodal in nature, where several modalities (such as the different contrasts of an MRI study, or paired CT and MRI acquisitions) describe the same underlying anatomy. Multimodal VAEs were proposed to learn a single shared latent space that jointly encodes all modalities, so that the model can reason from any subset of them, including the cases where some modalities are missing. Given *M* modalities $\left\{x_{1},\ldots ,x_{M}\right\}$, the goal is to approximate the joint posterior $q_{\phi }\left(z|x_{1},\ldots ,x_{M}\right)$ from the individual unimodal encoders $q_{\phi }\left(z|x_{m}\right)$.

The Multimodal VAE (MVAE) proposed by Wu and Goodman [22] combines the unimodal encoders using a product of experts (PoE):

(11) $$q_{\phi }\left(z|x_{1},\ldots ,x_{M}\right)\propto p\left(z\right)\prod_{m=1}^{M}q_{\phi }\left(z|x_{m}\right)$$

The main advantage of this formulation is that it handles missing modalities naturally, since a missing modality is simply dropped from the product without changing the structure of the model. The MMVAE proposed by Shi et al. [23] instead uses a mixture of experts (MoE):

(12) $$q_{\phi }\left(z|x_{1},\ldots ,x_{M}\right)=\frac{1}{M}\sum_{m=1}^{M}q_{\phi }\left(z|x_{m}\right)$$

which trades the sharp precision-weighted fusion of the PoE for a more robust cross-modal generation. Later, Sutter et al. [24] proposed MoPoE-VAE, which generalizes both approaches by taking a mixture over the products of experts of all modality subsets, so it keeps the advantages of both the PoE and the MoE. These multimodal formulations are the foundation for the multimodal and missing-modality segmentation methods that we discuss later in Section 8, where the shared latent space itself becomes the fusion mechanism for segmentation across modalities.

Table 3 summarizes the main characteristics of these VAE variants.

## 3. Roles of VAEs in Medical Image Segmentation

A VAE is not a segmentation architecture by itself like U-Net or other related architectures. Instead, the VAE architecture usually contributes to the segmentation task through its latent representation and the way it is used within the pipeline. Across the reviewed studies we have gone through, we identified five main roles that appear repeatedly and form the taxonomy used throughout this review.

Role A: Generative augmentation.

In this role, the VAE is used to create additional training data. It can generate synthetic medical images as it can also generate synthetic image–mask pairs using conditional variants of VAE. These synthetic samples are then combined with the original dataset and used to train a separate segmentation model, such as U-Net or ResUNet++, which then helps increase the amount of available data and can also improve the representation of classes that appear less often.

Role B: Latent representation integration.

In this role, the VAE is used as part of the segmentation network through its latent representation rather than being used for data generation. This provides the segmentation network more powerful settings as the VAE learns a structured representation of plausible anatomy, and can provide additional information that complements the local features extracted by the convolutional layers. For example through a shared decoder or by adding latent features to the skip connections of a U-Net, which can help the network when parts of the image are unclear, missing, or affected by low contrast.

Role C: Unsupervised anomaly localization.

Here, the VAE is trained using only the normal images, and when abnormal image is presented, the model usually reconstructs the normal structures more accurately than the pathological region as the model has only learned the appearance of healthy anatomy. The difference between the input and the reconstruction can then be used to highlight suspicious areas and produce an approximate localization of the anomaly. This makes it possible to identify lesions without requiring pixel level annotations during training, which can be useful when labeled examples are limited or when rare abnormalities are difficult to collect.

Role D: Multimodal latent fusion and missing-modality segmentation.

This role builds on the multimodal VAEs introduced in Section 2.3.6. Rather than supporting a single-modality backbone as in Role B, the shared latent space is used to combine information from several available modalities. Each modality can be encoded separately, and their representations are combined into a shared latent space that is used for segmentation. This allows the same model to continue producing the segmentation when one or more of the expected modalities are missing. In the studies reviewed here, this role is mainly applied to missing-sequence brain MRI segmentation.

Role E: Anatomical shape priors and segmentation quality control.

In this role, the VAE is trained on segmentation masks or anatomical representations to learn the distribution of realistic and plausible shapes. The learned representation can then be used to detect anatomically unrealistic segmentation masks, correct implausible predictions, or identify segmentation failures. This role differs from Role B, where the VAE contributes to the segmentation network through latent representation learning or regularization; here, the VAE is used primarily as an anatomical prior or as a quality-control mechanism for the predicted segmentation.

These roles are not mutually exclusive, and some methods can combine two or more of them. For example, a VAE can be used to generate additional training samples (Role A) while another variational component is integrated within the segmentation network for latent representation learning or regularization (Role B). Similarly, a multimodal model (Role D) may use a shared latent representation to handle missing modalities while still producing the segmentation. In the remainder of the review, the role or roles fulfilled by each method are indicated in the summary tables.

## 4. VAEs for Segmentation in Radiography

Chest X-rays are among the most commonly performed medical imaging examinations, making them a natural target for VAE-based methods. Several challenges characterize this domain, including the scarcity of labeled segmentation masks, severe class imbalance for rare pathologies, and particularly relevant to segmentation heavy opacification in pathologies such as COVID-19 or congestive heart failure that obscures lung regions and degrades the performance of conventional segmentation networks. VAEs have been applied to address these challenges through several strategies: latent representation integration with U-Net for robustness to opacification, multi-scale VAE fusion with attention for improved lung boundary delineation, two-stage pipelines that first enhance the image (via a VAE) and then segment it, generative augmentation to enlarge training sets for segmentation networks, and explainable saliency maps that approximate pathology localization.

### 4.1. Lung Segmentation Under Heavy Opacification

Selvan et al. [25] proposed a VAE-based approach for lung segmentation in chest X-rays with high pulmonary opacity, including COVID-19 cases. The main challenge is that extreme opacification obscures lung regions, which makes segmentation difficult even for trained models. The architecture combines a U-Net and a variational autoencoder that share the same decoder. The variational encoder maps input images to a low-dimensional latent space (dimension 8), and samples from this space are concatenated with the U-Net encoder features before decoding the segmentation. The model was trained on 528 normal CXRs from two datasets, Shenzhen and Montgomery [26,27], with extensive data augmentation. When tested on the same 30 diverse, expert-annotated CXRs with high opacification from COVID-19 repositories, the proposed model achieved a Dice score of 0.85 and accuracy of 0.88, which outperformed the modified U-Net (Dice 0.82; accuracy 0.87) under the same block-masking and diffused-noise augmentation. This work demonstrates the effectiveness of VAE latent representations (Role B) for handling missing or corrupted regions in medical image segmentation.

Cao and Zhao [28] proposed a lung segmentation algorithm that combines a Fusion VAE (FVAE) with a three-terminal attention mechanism. Compared to previous approaches that integrate VAE only at the network bottleneck, this approach is different in that it introduces VAE modules at each encoder layer, fusing convolutional features with VAE latent representations to capture both local details and high-level semantic information such as thorax symmetry and lung contours. The three-terminal attention mechanism enhances skip connections by combining channel attention (via squeeze-and-excitation modules) with spatial attention guided by multi-scale features. Training on the Montgomery and Shenzhen datasets [26,27] with data augmentation, the method was evaluated on the challenging SNIH dataset [29] (100 abnormal CXRs with severe opacities) and JSRT [30] dataset (247 images). The proposed approach achieved Jaccard scores of 0.882 and 0.920 on SNIH and JSRT, respectively, which outperformed the baseline U-Net (0.862 and 0.892, respectively) on the same datasets in Cao and Zhao’s evaluation. This is again an example of Role B: VAE latents are embedded inside a segmentation backbone, but here at every encoder level rather than only at the bottleneck.

The shared encoder design underlying these methods has also been applied to related quantification tasks rather than to segmentation itself. (Although its primary task is severity quantification rather than segmentation, the same shared-encoder design appears in the work of Liao et al. [31], who proposed a semi-supervised VAE that jointly trains an encoder, a decoder, and an ordinal regressor to estimate pulmonary edema severity from chest X-rays of congestive heart failure patients. Labeled and unlabeled images pass through a shared encoder; the decoder is trained on all images, whereas the regressor is trained only on the labeled ones, reaching an RMS error of 0.66 and a Pearson correlation coefficient of 0.52. In VAE-augmented segmentation networks, this regressor is simply replaced by a segmentation head).

### 4.2. Two-Stage Pipelines: VAE-Based Enhancement Followed by Segmentation

Ibrahim et al. [32] proposed a two-stage approach for lung segmentation combining VAE-based image enhancement with ResUNet++ segmentation. The first stage employs VAEU-Net, an architecture that concatenates features from a VAE encoder with a U-Net encoder to perform cross-domain mapping for artifact removal and image enhancement. The VAE maps degraded CXR images to a low-dimensional latent space, and the combined features from both encoders are decoded through a shared decoder to produce enhanced images. The second stage utilizes ResUNet++, which integrates residual blocks, squeeze-and-excitation attention, Atrous Spatial Pyramidal Pooling (ASPP), and attention mechanisms for robust lung segmentation with limited training data. Trained on the JSRT [30] dataset, the approach achieved Dice scores of 0.990 and 0.985 on JSRT and NLM-MC, respectively. Furthermore, the VAEU-Net enhancement stage improved the Dice score on the COVID-19 and lung opacity datasets by 1.65% and 3.63%, respectively, in the study’s evaluations. This work illustrates how VAE-based image enhancement can be used as a preprocessing stage before a dedicated segmentation backbone to improve performance under domain shift.

### 4.3. VAE–GAN Image Synthesis as a Related Application

Rguibi et al. [33] explored the combination of a VAE and a GAN for generating synthetic chest X ray images. The VAE first learns a compact probabilistic representation of the images, while the discriminator encourages the decoder to produce outputs that are closer to real radiographs. The model was trained on 1341 images selected from the NIH ChestX ray dataset [34], and the quality of the generated images was evaluated using the Inception Score and the Fréchet inception distance. The reported Inception Score increased from 1.104 at the beginning of training to 1.843 at the final epoch, close to the value of 1.854 obtained for the real images, while the reported FID decreased strongly during training. This study focused on synthetic data generation and did not perform segmentation; however, the authors mentioned that the generated data could later be used for tasks such as segmentation. Hence, this study demonstrates the potential of combining the structured latent space of a VAE with the image realism provided by adversarial training, rather than showing a direct segmentation improvement.

### 4.4. Explainable Pathology Localization (Approximate Segmentation)

Iglesias et al. [35] proposed MOC-VAE, which combines VAE reconstruction with classification and uses a multi-objective genetic algorithm to find minimal changes in the latent space that modify the predicted class. These changes are then decoded into saliency maps that highlight pathology-related regions. Evaluated on the PadChest dataset, these maps correctly identified the pathological region in 80.56% of the cases. However, these maps are not pixel-level segmentation masks, and the method does not follow the reconstruction-based anomaly localization mechanism defined in Role C. Therefore, we consider this work as a weak localization and explainability application related to segmentation rather than as a Role C method.

### 4.5. Breast Tumor Segmentation in Mammography

Balaji [36] has proposed a 3D Connected-UNets architecture for breast tumor segmentation, where two U-Net architectures are connected through additional skip connections and a VAE branch is added to the network during training. The VAE reconstructs the input image from the shared representation, which provides an additional constraint on the learned features and helps regularize the segmentation network when the amount of training data is limited. The initial segmentation is further refined using a fully connected 3D conditional random field and connected-component analysis to reduce segmentation noise. The datasets used in this study were INbreast [37] and CBIS-DDSM [38], together with a private mammography dataset. On the public datasets, the authors reported tumor true-positive rates of 95% on CBIS-DDSM and 98% on INbreast, while the corresponding true-negative rates were 90% and 92%, respectively. This work represents Role B, since the VAE is integrated into the segmentation architecture mainly to regularize the shared representation during training.

Table 4 displays the main works presented in this section. In chest X-ray imaging, VAEs are used mainly to improve lung segmentation under difficult conditions such as heavy opacification. The direct role-based applications presented here mainly correspond to latent representation integration (Role B), where the VAE is incorporated into the segmentation architecture. Other works use VAEs for image enhancement before segmentation, image synthesis, or explainable pathology localization. These related applications are presented separately because they do not directly fit the definitions of the five roles used in our taxonomy.

## 5. VAEs for Segmentation in Computed Tomography

Computed Tomography (CT) is a medical imaging technique that uses X-rays taken from multiple angles to produce cross-sectional (slice) images, used for detecting and characterizing lesions in organs such as the lungs, liver, and brain. The analysis of CT images has some challenges such as the volumetric (3D) shape of the data, scanner-dependent variations and the need for large annotated datasets. For segmentation, VAEs address these difficulties through the roles introduced in Section 3, and in this section they appear mainly for probabilistic segmentation and latent representation integration, unsupervised anomaly localization, image enhancement before segmentation, and anatomical shape priors that check the predicted masks.

### 5.1. Probabilistic Segmentation and Annotation Uncertainty

One of the direct uses of variational models in CT segmentation is to represent the variability between different possible annotations. Kohl et al. [39] proposed the Probabilistic U-Net, which combines a U-Net with a conditional VAE to learn a distribution over plausible segmentation masks rather than producing only one deterministic prediction. During the training, the posterior network receives both the image and its corresponding segmentation mask, while the prior network learns the latent distribution directly from the image. During inference, different samples from this distribution can therefore produce different plausible segmentation masks. The approach was evaluated on lung abnormalities from the LIDC-IDRI dataset [40], where lesions were independently annotated by multiple radiologists. The model was able to reproduce different plausible segmentation variants together with their occurrence frequencies. This represents a direct integration of variational latent modeling into the segmentation framework (Role B), while also providing a way to explicitly represent annotation uncertainty. Building on the Probabilistic U-Net, Baumgartner et al. [41] have proposed PHiSeg, a hierarchical probabilistic segmentation framework that represents segmentation distribution at multiple resolutions. Instead of introducing a single latent variable, separate variational latent representations are learned at different levels of the network, where the lower-resolution variables capture coarse variations while the higher-resolution ones progressively refine the segmentation. The method was evaluated on LIDC-IDRI lung CT [40] and an in-house prostate MRI dataset with multiple expert annotations. On LIDC-IDRI, the five-level PHiSeg achieved a generalized energy distance of 0.225 compared with 0.239 for the Probabilistic U-Net, while also providing better agreement between predicted uncertainty maps and the variability between expert annotations (normalized cross-correlation 0.845 versus 0.775). Both models were evaluated with all available expert annotations and 100 generated samples per model; these scores refer to LIDC-IDRI, not the separate prostate MRI evaluation. This represents another direct use of variational latent modeling for probabilistic segmentation (Role B), with the hierarchical latent space allowing variation between plausible masks to be represented at different spatial resolutions. Valiuddin et al. [42] have further investigated latent-density models for probabilistic medical image segmentation, focusing particularly on the way the latent space is used to represent aleatoric uncertainty. The authors showed that the latent representation learned by the Probabilistic U-Net can become sparse, where only a limited number of latent dimensions contribute effectively to the generation of different segmentation hypotheses. To address this limitation, they introduced mutual-information maximization together with an entropy-regularized Sinkhorn divergence in the latent space, which encourages the different latent dimensions to carry more useful information and results in a more homogeneous representation. The method was evaluated on the LIDC-IDRI dataset [40], as well as the pancreas, pancreatic-lesion, prostate and brain-growth tasks from the QUBIQ dataset [43], covering both CT and MRI segmentation problems with multiple annotations. Compared with the original Probabilistic U-Net and a normalizing-flow extension on the LIDC-IDRI and QUBIQ tasks, the proposed approach improved the modeling of the segmentation distributions, with performance gains reaching up to 11% according to the Hungarian-matched IoU evaluation in Valiuddin et al.’s comparisons. This work therefore represents Role B, where the variational latent space is used to model multiple plausible segmentations and the variability between annotations.

### 5.2. Unsupervised Anomaly Localization

Anomaly detection is one of the most studied roles for VAEs in CT. In this application the model is usually trained on normal scans alone, and then during the reconstruction, the model reconstructs healthy anatomy well but reproduces unfamiliar, diseased structures poorly, and the residual between the input and its reconstruction marks the abnormal region, producing a coarse segmentation with no annotated lesions.

Uzunova et al. [44] proposed an unsupervised method for pathology detection that is based on a conditional variational autoencoder trained only on healthy medical image patches (see Figure 4). Their framework divides each 2D or 3D image into overlapping patches. Additionally, they used also the normalized spatial position of each patch as a condition so that the model learns the expected appearance of healthy tissue at each anatomical location. During inference, every patch is encoded and reconstructed independently, and then, its anomaly score is calculated by multiplying the reconstruction error by the distance between its latent representation and the healthy latent distribution. The patch-level scores are then projected back to their original locations and averaged in overlapping regions to generate a full-image pathology heatmap, which can be thresholded to obtain a rough lesion mask. The method was evaluated using brain MRI from BraTS [45] and LPBA40 [46], as well as on thoracic CT images containing different lung pathologies, including lung cancer and fibrosis, where it qualitatively localized abnormal regions despite the absence of segmentation ground truth. It achieved a Dice of 0.55 and an AUC of 0.95 on 2D BraTS, while multimodal 3D MRI reached a Dice of 0.50. However, the method does not perform well on complex data as it provides only coarse localization, and requires approximately aligned images.

The same principle extends naturally to volumetric CT, and two studies have illustrated its applicability across different clinical settings. Ogawa et al. [47] proposed an unsupervised 3D-VAE framework for detecting inner-ear malformations from temporal-bone CT scans. The dataset used contains 6776 inner-ear volumes; 6663 are normal and 113 are malformed cases. For unsupervised learning, 6550 normal volumes were used for the training and the validation, while all 113 malformations and 113 age-matched normal volumes were kept for testing. After resampling, intensity clipping, left–right standardization, and semiautomatic cropping, each complete 48×48×40 inner-ear volume was reconstructed by the 3D-VAE. During inference, voxel-wise differences between the input and reconstructed volumes were thresholded to generate an anomaly map, while the ratio of highlighted voxels was used to classify each case as normal or malformed. Their method achieved an AUC of 0.99, 95.6% accuracy, 99.1% sensitivity, and 92.0% specificity, while fully localizing abnormal regions in 93% of malformation cases. They also tested their approach against a supervised 3D-ResNet. Although the 3D-ResNet achieved 99.8% specificity and 90.3% sensitivity using 10-fold cross-validation, its scores cannot be directly ranked against the 3D-VAE’s 92.0% specificity and 99.1% sensitivity on 113 malformations and 113 held-out normal cases because the protocols differed. Its LIME [48] maps localized the complete abnormal anatomy less consistently in Ogawa et al.’s evaluation. Chung and Choi [49] in another work have investigated whether a 3D-VAE could detect abnormal extraocular muscle enlargement associated with thyroid eye disease using a relatively small orbital CT dataset. They retained 233 contrast-enhanced CT scans from 262 collected examinations and separated them into 465 individual orbital volumes, including 358 normal and 107 abnormal orbits. The model was trained only on 276 normal volumes, while 58 normal and 96 abnormal volumes were used for validation, and 24 normal and 11 abnormal volumes were reserved for testing. Before training, each orbit was extracted from coronal CT slices, left-sided images were flipped to match the orientation of right-sided images, and all volumes were resized to 64×64×64 voxels. The authors also adjusted the voxel intensities to emphasize the extraocular muscles and reduce the influence of the high-intensity orbital bones, since the model initially focused more on bone contours than on the small target muscles. The encoder compressed each orbital volume into a latent representation of eight dimensions, and the decoder reconstructs the complete volume using transposed convolutions and an upsampling layer. The model was trained for 100 epochs using only the normal volumes, and the loss is the combination of the voxel-wise reconstruction error plus the KL-divergence, while the squared voxel-level differences between the input and reconstruction were visualized as heatmaps to localize enlarged muscles. Using ROC analysis, they selected a threshold of 21,636 on the validation data, and their model achieved an AUROC of 0.801, 77.1% accuracy, 79.2% sensitivity, 72.7% specificity, and an F1-score of 0.667 on the test set.

COVID-19 produced the most direct quantitative benchmark for a VAE used as a segmenter. Zhou et al. [50] proposed WVALE, a weakly supervised method for locating COVID-19 lung infections in CT slices. A small VAE was trained only on healthy images, using both the original slice and an altered version created by doing modifications on the original slice like shifting, removing a lung patch, or mixing it with another healthy image which helps the model learn the usual appearance of healthy lungs. During testing, the model generates heatmaps from its latent features and combines them into one infection map, which is then converted into a lesion mask or used as an automatic label for training segmentation models. The study used data from 66 COVID-19 patients, 66 healthy controls, and a separate set of 100 manually segmented CT images, and their method achieved a Dice score of 0.559, 66.7% sensitivity, and 96.2% specificity for its lesion masks. Its generated labels improved U-Net++ Dice from 0.643 to 0.791 on the same annotated CT segmentation set in Zhou et al.’s five-fold cross-validation. However, their approach sometimes highlights normal blood vessels as abnormal, and some annotated images were still needed to choose the final threshold.

Across these studies, the architecture varies while the principle stays almost the same: a VAE is trained only on normal anatomy, and a large reconstruction error or latent-space deviation flags the abnormality at test time.

Figure 5 shows the main architectural variants, which differ only in how the input is conditioned, how the latent space is represented, and how the anomaly is read out.

### 5.3. Tumor Modeling and CT Enhancement for Downstream Segmentation

VAEs have also been applied to feature extraction and tumor progression modeling in support of segmentation workflows. Li et al. [52] developed a 3D beta-VAE to learn useful representations of lung lesions from CT scans without using clinical labels during training. The model was trained on lesion patches extracted from the LIDC-IDRI, LNDb, and Stanford radiogenomics datasets, after resampling the scans to 1×1×1 mm resolution and centering each patch around a radiologist-provided lesion location. The model contains four 3D convolutional blocks that compressed each patch into a 1024-dimensional latent representation, and then the decoder used this representation to reconstruct the original lesion volume. For their experimentation, the authors have used three datasets, which are LIDC-IDRI [40], LNDb [53], and the Stanford NSCLC radiogenomics dataset [54], and their model has achieved as results an SSIM of 0.774, a PSNR of 26.10, and an MSE of 0.001. The features that were learned during training has captured lesion size, which allows then the authors to generate enlarged or reduced synthetic nodules. Additionally, these features were used further with prediction models like XGBoost to predict clinical properties such as KRAS and EGFR mutations, pathological stages, and lymphovascular invasion, with performance generally comparable to radiomics and supervised CNN models. In another work, Xiao et al. [55] proposed their framework CRVA (conditional recurrent variational autoencoder) (see Figure 6) to predict the future appearance of lung tumors from follow-up CT scans using a combination of longitudinal data from the National Lung Screening Trial (NLST) [56] and Shanxi Provincial Cancer Hospital, using 880 patients in the final experiments. After image registration and manual tumor segmentation, each lesion was cropped into a 64×64 region of interest. The authors in their approach used the tumor images from the first two examinations and the framework’s job is to predict the third tumor growth. The first two tumor images are given in the first stage to the encoder to encode them into latent vectors, and recurrent units learned their changes over time to predict the tumor state at the third visit. As the model is conditional, they have used additional data for each patient including smoking and alcohol use, medical and family cancer history, and occupational background to make the prediction more personalized, and total-variation regularization was added also to reduce blurry outputs. In the longitudinal tumor-growth prediction evaluation, their method achieved a Dice score of 82.49%, precision of 82.22%, and recall of 79.89%.

A closely related use of VAEs is CT image enhancement in radiation therapy workflows, where high-quality images are required for accurate organ-at-risk segmentation. Joseph et al. [57] proposed a Feature-Embedded VAE (FE-VAE) that improves Cone Beam CT (CBCT) images used in Image Guided Radiation Therapy, combining a MultiResUNet-based feature extractor with a perceptual loss to synthesize high-quality Fan Beam CT images from lower-quality CBCT scans without additional radiation exposure. The enhanced images are directly usable as input to downstream segmentation networks.

### 5.4. VAEs as Shape Priors for Segmentation Quality Control

The studies above all encode the CT image itself. A complementary line of work instead encodes the *segmentation mask*, using the VAE to learn the distribution of plausible organ shapes. Because a segmentation carries far less variation than the underlying image, this distribution can be learned reliably, and any mask that deviates from it is, by construction, suspicious.

Sandfort et al. [58] have proposed an unsupervised framework based on VAEs to detect incorrect liver, kidney, and spleen segmentations in abdominal CT scans. Three separate 3D U-Nets were first trained on datasets containing 141 liver, 51 spleen, and 66 kidney cases, then applied to 12,495 CT examinations to generate automatic organ masks. The authors then trained one 3D VAE per organ directly on these automatic outputs, assuming that most of them were reasonably correct and therefore represented the usual organ shape. Each binary mask was resized to 64×64×64, compressed into a 10-dimensional latent vector, and reconstructed by the decoder. The Dice similarity between the original U-Net mask and the VAE reconstruction was then used as a quality score rather than as a conventional segmentation score against manual ground truth. For evaluation, a physician independently reviewed 2510 masks for each organ and labeled them as acceptable or problematic, allowing the reconstruction scores to be assessed using ROC analysis. The method achieved AUCs of 0.94 for liver, 0.90 for kidney, and 0.81 for spleen, outperforming conventional outlier detectors in most of Sandfort et al.’s comparisons on the same physician-reviewed CT masks. The detected difficult cases were also used in an active-learning experiment, reducing the spleen segmentation error rate from 7.1% to 5.7%.

What makes this study interesting is that the VAE is not used to create the segmentation mask, but to check whether an already generated mask is reliable. By learning the common shapes of organs, the model can recognize unusual masks through poor reconstruction. The reconstruction error therefore becomes an automatic score for quality-control, which helps then to identify segmentation failures before they are used in clinical practice. This work therefore represents Role E, where the VAE is used as an anatomical shape prior and as a quality-control mechanism for segmentation masks.

A different use of the VAE as an anatomical shape prior was proposed by Wang et al. [59] for annotation-efficient medical image segmentation. Their framework uses a CycleGAN to generate pseudo-segmentation labels from medical images that is not annotated, while a VAE-based discriminator is trained using auxiliary segmentation masks to learn the distribution of plausible anatomical shapes. The discriminator then provides a high-level shape constraint on the generated pseudo labels, and its feedback is also used to calibrate the generator before a separate segmentation network is trained using a noise-robust iterative learning strategy. The framework was evaluated across several tasks, including optic disc, fetal head, lung and liver segmentation. For liver segmentation, the method was evaluated on the CHAOS dataset [60], where CT images were segmented using auxiliary liver masks obtained from MRI. The VAE-based discriminator improved the mean Dice score of the pseudo-label generator from 0.853 to 0.869 in Wang et al.’s ablation on the same CHAOS CT liver test set, while the final segmentation model reached a Dice score of 0.933; the latter is a separate result from the pseudo-label comparison. This work can therefore be classified as Role E, where the VAE learns anatomical shape information and uses it to constrain the generation of plausible segmentation masks. Yao et al. [61] have proposed an unsupervised domain adaptation framework for pancreas segmentation based on a VAE anatomical shape prior. In the first stage, the VAE is trained on the segmentation masks from the source domain in order to learn the distribution of plausible pancreas shapes, and this learned representation is then used during the adaptation to the target domain. Since no annotations are available for the target images, a teacher–student framework is used to generate the pseudo labels, while the reconstruction loss of the VAE is applied to encourage these predicted masks to remain close to the anatomical shape distribution that is learned from the source data. The authors have evaluated their approach using the NIH Pancreas-CT dataset [62] as the source domain, while the Medical Segmentation Decathlon [63], Synapse [64], and two in-house pancreas CT datasets were used as target domains. The proposed method achieved mean Dice scores of 0.757 on MSD, 0.787 on Synapse, 0.826 on the in-house IV-contrast dataset, and 0.745 on the oral-contrast dataset, showing better segmentation performance than applying the source-only 3D U-Net to the same target splits and with the same preprocessing (0.678, 0.745, 0.798, and 0.702, respectively) in Yao et al.’s evaluation. This work therefore represents Role E, where the VAE is used to learn a distribution of plausible anatomical shapes and then constrains the segmentation predictions during domain adaptation.

Taken together, the studies reviewed in this section show that VAEs support CT segmentation in different ways: by integrating latent representations or regularizing segmentation models (Role B), by localizing pathology without labels (Role C), by supporting related tasks such as tumor modeling and image enhancement before segmentation, and by acting as anatomical shape priors for segmentation quality control (Role E). Table 5 consolidates these works according to their relationship with segmentation, together with the VAE variant, the task, the datasets used, and the reported results.

## 6. VAEs for Segmentation in MRI

MRI is one of the most used imaging modalities in medicine, especially for neurological, cardiac, and soft-tissue analysis, and its segmentation is an important step in many clinical tasks such as brain tumor delineation and the measurement of cardiac function. What makes MRI different from the modalities discussed before is that a single examination usually produces several sequences at the same time, for example T1, contrast-enhanced T1, T2, and FLAIR in brain imaging, and each sequence shows the tissues in a different way. The fusion of these sequences, and the case where some of them are missing, is an important part of unified and multimodal segmentation, so we treat it separately in Section 8. In this section, we focus only on how VAEs are used for MRI segmentation within a single modality.

In this modality, VAEs are used in three main ways: first, a VAE branch is added to a supervised segmentation network and reconstructs the input image to regularize the shared encoder, and this design gave the winning method of the BraTS 2018 challenge and is still commonly used in brain tumor pipelines. Second, a VAE is trained only on healthy brain scans, and the pathology is then localized from its reconstruction error, which is the same unsupervised anomaly principle already seen in CT but this approach here is applied to a large number of brain MRI works. Third, outside the brain, VAEs are used as anatomical shape priors and as representation learners for cardiac and other anatomies, where producing an anatomically plausible shape is as important as the overlap score. We follow these three directions in the rest of this section.

### 6.1. VAE-Regularized Segmentation Networks for Brain MRI

The most influential use of VAEs in MRI segmentation is as a regularizer for a supervised segmentation network. Myronenko [65] introduced this idea in the method that won the BraTS [45] 2018 challenge (Figure 7). The input to this network is a 3D volume formed by stacking the four BraTS sequences (T1, contrast-enhanced T1, T2, and FLAIR), and the network follows an encoder–decoder structure built from residual blocks with group normalization. A large asymmetric encoder progressively downsamples the volume and extracts deep features, and a smaller segmentation decoder upsamples them back to the three nested tumor sub-regions, the whole tumor, the tumor core, and the enhancing tumor, each predicted with a sigmoid output. Because the annotated training set was small, a second branch, a variational autoencoder, was attached to the end of the encoder. This branch compresses the encoder features into a low-dimensional Gaussian latent, samples from it, and reconstructs the entire four-channel input volume. Reconstructing the input forces the shared encoder to keep a smooth and complete representation of the image, and not only the features that are directly useful for the segmentation, which reduces overfitting and improves generalization. The VAE branch is used only during training and is removed at inference, so it adds no computational cost at test time. The total loss combines a soft Dice term for the segmentation with an $L_{2}$ reconstruction term and a KL-divergence term that keeps the latent close to a standard normal prior. Trained with a large 3D crop of size 160×192×128 and an ensemble of models, the method reached Dice scores of 0.766 for the enhancing tumor, 0.884 for the whole tumor, and 0.815 for the tumor core on the BraTS 2018 test set.

The following editions of the challenge moved towards cascaded designs, where a first network produces a coarse segmentation and a second one refines it. Jiang et al. [66] won BraTS 2019 [45] with such a cascade, but they replaced the reconstruction branch by a second segmentation decoder used only during training, so their regularization is no longer variational.

Lyu and Shu [67] kept both ideas together and placed a real VAE branch inside a cascade. In the first stage, the network is the asymmetrical U-Net with a VAE branch of Myronenko [65], and it takes a cropped volume of the four MRI sequences of size 4×160×192×128 and outputs the three tumor sub-regions. The VAE branch reduces the encoder endpoint to 256 values with a fully connected layer, where these values represent 128 means and 128 standard deviations of Gaussian distributions, then a vector of size 128 is sampled from them and mapped back to reconstruct the input volume. In the second stage, the coarse segmentation maps of the first stage are concatenated with the original MRI images and given to a second network, which keeps its own VAE branch and adds attention gates so that the model focuses on the relevant regions instead of the whole volume. This second stage is also trained on an expanded dataset formed from the outputs of the first stage, and because the VAE regularization is used in both stages, the two encoders are protected from overfitting at the same time. This work has been done using the BraTS 2020 validation set [45] and their method has reached Dice scores of 0.904 for the whole tumor, 0.835 for the tumor core, and 0.796 for the enhancing tumor, and on the test set 0.873, 0.836, and 0.821 respectively, which placed it among the top ten teams of the challenge. This work has demonstrated that the VAE can be combined with other attention mechanisms and can also be a good and useful regularizer even inside a complex pipeline with multiple stages.

Tang et al. [68] proposed a VAE-regularized 3D MultiResUNet for brain tumor segmentation. The model combines MultiRes blocks with a VAE branch connected to the encoder. While the main decoder predicts the tumor segmentation, the VAE branch reconstructs the input MRI and is optimized using reconstruction and KL-divergence losses. This auxiliary branch is used to regularize the encoder and reduce overfitting. The model was trained on the BraTS 2020 dataset [45] using the four MRI modalities and achieved Dice scores of 0.703, 0.893 and 0.790 for enhancing tumor, whole tumor and tumor core, respectively. This approach therefore represents Role B, where the VAE is integrated into the segmentation architecture as a regularization mechanism. Pham et al. [69] have proposed SegTransVAE, a hybrid architecture that combines CNNs, a Transformer and a VAE branch for medical image segmentation. The convolutional encoder is first used to extract local features from the input, while the Transformer operates on the deeper representations in order to capture longer-range dependencies that can be difficult to model using convolution alone. In parallel, a VAE branch is connected to the shared representation and reconstructs the input image, with the reconstruction and KL-divergence losses being optimized together with the segmentation Dice loss, so that the latent representation remains regularized during training. The authors evaluated their method on the BraTS 2021 dataset [45] for brain tumor MRI segmentation and on the KiTS19 dataset [70] for kidney and kidney tumor CT segmentation. On BraTS 2021, SegTransVAE achieved Dice scores of 85.48%, 90.52% and 92.60% for the enhancing tumor, whole tumor and tumor core, respectively, while on KiTS19 it achieved average Dice scores of 95.28% for the kidney and 66.31% for the tumor. This work therefore represents Role B, where the VAE is integrated into a larger hybrid segmentation architecture as a regularization mechanism, while the Transformer is used to model more global relationships in the image.

A different way to use a VAE for supervised segmentation is to generate the training data instead of regularizing the encoder. Kebaili et al. [71] proposed a discriminative Hamiltonian VAE (dHVAE) for tumor segmentation when only a few annotated scans are available, and their architecture is shown in Figure 8. The image and its mask are concatenated channel-wise and given together to the encoder, so the model learns their joint distribution and produces a complete image–mask pair in a single pass, instead of conditioning on a mask that would have to be obtained first. In the latent space, a Hamiltonian VAE [72] replaces the usual single sample by a short Hamiltonian Monte Carlo chain, where the latent code is treated as a particle refined by a few leapfrog steps, which gives a more accurate posterior when the training set is very small. Two regularization terms were added to correct the blurriness of VAE outputs which are: a discriminative term from a PatchGAN discriminator and a perceptual term computed on a pretrained VGG16 network [73], and the model works slice by slice because training a 3D generator on a few volumes is not realistic. The method was evaluated using the two datasets: BraTS 2021 [45] for MRI and HECKTOR 2022 [74] for PET. The generated image–mask pairs reached an FID of 88.3 and for segmentation, adding 2000 synthetic pairs raised the Dice score of a U-Net from 0.607 to 0.724 on BraTS and from 0.454 to 0.594 on HECKTOR, and nnU-Net reached 0.745 and 0.610 after the same augmentation.

### 6.2. Probabilistic Segmentation and Inter-Observer Variability

Liu et al. [75] have proposed a variational inference framework for medical image segmentation that explicitly models the variability between the annotations provided by different experts. Instead of learning only one deterministic segmentation for each image, the model uses a latent vector to represent the differences between the possible annotations, where different samples from this latent space can produce different plausible segmentation masks for the same input image. The framework is composed of an encoder, a decoder that reconstructs the input image, and a segmentation network, and the whole model is trained using the segmentation loss together with the reconstruction and KL-divergence losses. The authors evaluated their approach on the brain-growth MRI task from the QUBIQ dataset [43], where each image was annotated by seven different experts. The dataset contains 34 subjects for training and 5 for validation in this task. By averaging seven generated segmentation predictions on the five-subject QUBIQ brain-growth validation split, the model achieved a continuous Dice score of 0.537, compared with 0.504 for the ESULR baseline and 0.508 for Bayesian dropout on the same split. This work therefore represents Role B, where the variational latent representation is directly used to model the distribution of plausible segmentations and the variability between different expert annotations.

### 6.3. Weakly and Minimally Supervised Segmentation with Anatomical Priors

Yu et al. [76] have proposed the Segmentation Auto-Encoder (SAE), which is a variational framework for brain MRI segmentation with minimal supervision. Their approach is different from conventional VAEs because, instead of using a continuous latent vector, it treats the segmentation map itself as a discrete latent representation. A 3D U-Net first receives the input MRI and predicts a probabilistic segmentation map, which represents the different anatomical regions of the brain. This predicted segmentation is then given to a decoder whose job is to reconstruct the original MRI image. In this way, the model is forced to encode the important anatomical information of the input directly into the segmentation map, since this map must contain enough information for the decoder to reconstruct the corresponding image.

The predicted segmentation is additionally constrained using an anatomical prior that represents the expected distribution of the brain structures. An interesting aspect of their approach is that this prior does not necessarily require paired image–mask training samples and can be obtained even from a single unpaired atlas. The KL-divergence is then used to keep the predicted segmentation distribution close to this anatomical prior. The authors also introduced a Markov Random Field to model the spatial relationships between neighboring labels and reduce anatomically inconsistent predictions. They evaluated their approach on 38 T1-weighted brain MRI scans, and the best SAE configuration achieved a Dice score of 80.54% and an HD95 of 2.68 mm, compared with 84.60% Dice for the fully supervised U-Net on the same test subjects in each of five repeated 30-subject training and eight-subject test splits. Despite the fact that the fully supervised model remained more accurate, the SAE model achieved close performance without paired training masks. This work shows that the variational autoencoder can be used for other tasks rather than reconstruction or latent regularization, but also it can be used to directly constrain the segmentation itself using anatomical knowledge when annotated data are limited. Zhou et al. [77] have proposed EFS-MedSeg, a few-shot medical image segmentation framework that combines supervised segmentation with self-supervised VAE representation learning. Their method requires only two labeled atlases together with a small number of unlabeled images, and the labeled atlases are first augmented using a 3D random regional switch strategy to increase the variability of the training samples. In parallel, the VAE is used to reconstruct the unlabeled images, where a weighted reconstruction mechanism gives more importance to regions that are more difficult to segment. The authors also introduced a self-contrastive module that uses anatomical structure information to improve the learned features and produce more consistent segmentation boundaries. The method was evaluated on the OASIS brain MRI dataset [78], the BCV abdominal CT dataset [64], an in-house BCH abdominal CT dataset, and the VerSe spine CT dataset [79]. Compared with the second-best methods in Zhou et al.’s two-atlas few-shot evaluation, EFS-MedSeg improved the Dice score by 1.4% on OASIS, 9.1% on BCV, and 1.1% on BCH, while also showing competitive performance on the VerSe dataset in that study. This work represents Role B, where the VAE is used for self-supervised representation learning to improve segmentation when only a very limited amount of labeled data is available.

### 6.4. Unsupervised Anomaly Localization in Brain MRI

Brain MRI is the modality where unsupervised anomaly localization with VAEs has been studied the most. The principle is the one already described for CT in Section 5: the model is trained only on healthy scans, so it reconstructs normal anatomy well and fails on the lesions it has never seen, and the residual between the input and its reconstruction gives a segmentation without any lesion annotation. Two things make brain MRI well suited to this idea. Large collections of healthy brain scans are available and the second thing is that on FLAIR images the lesions appear bright against the normal tissue, so the reconstruction error is easy to read.

The reference work in this direction is not a new model but a comparison. Baur et al. [80] noticed that the published methods could not really be compared with each other, because each one was evaluated on a different dataset, on a different pathology, at a different image resolution, and with its own architecture. They therefore re-implemented the main models inside a single unified architecture and evaluated them under the same conditions, on several brain MRI datasets covering multiple sclerosis lesions and glioblastoma. The comparison is built around VAEs and their variants: the plain VAE with a dense and with a spatial bottleneck, the context-encoding VAE [81], the Gaussian-mixture VAE with and without an iterative restoration step, the constrained adversarial autoencoder [82], and two hybrids that add an adversarial loss, AnoVAEGAN and f-AnoGAN, with plain autoencoders kept as baselines.

Several conclusions come out of this study. The first is that the rankings of the compared models differed between the multiple-sclerosis and glioblastoma datasets in Baur et al.’s brain MRI benchmarks. The bottleneck design also matters, and the dense variants outperformed their spatial counterparts in Baur et al.’s tests using the same architecture and image resolution. The two adversarial hybrids gave much sharper reconstructions than the VAEs tested, but they modeled the healthy distribution so well that the reconstruction was sometimes anatomically different from the input, so AnoVAEGAN was only comparable to the VAE on the multiple-sclerosis datasets even if it performed better on the glioblastoma dataset in that study. The most general conclusion is that none of the models reconstructs a truly healthy version of a pathological input, so the quality of the reconstruction is not what separates a good method from a bad one. What matters is the gap between the reconstruction-error statistics of normal tissue and those of abnormal tissue, and the authors identify this gap as the best indicator of performance. They also show that the results depend strongly on the pathology, that the methods are sensitive to domain shift when the images come from another scanner or another site, and that the amount of healthy training data has a direct effect on the final accuracy. This study is the natural starting point of this subsection, and the works described below can be read as different attempts to widen that gap between normal and abnormal reconstruction errors.

The first way to widen it is to change the anomaly score itself. Zimmerer et al. [81] observed that the reconstruction error is a weak score for two reasons: it ignores the internal representation that the model used to produce the reconstruction, and its values are not really comparable from one sample to another. Their context-encoding VAE (ceVAE) answers both points. On the architecture side, a context-encoding task is added to the VAE, where parts of the input are hidden and the network has to fill them in, which forces it to learn the high-level structure of the brain instead of copying the input pixel by pixel, a behavior that standard VAEs often fall into. Using the BraTS 2017 [45] and ISLES 2015 [83] datasets, the ceVAE reached AUROCs of 0.95 and 0.89, respectively, above the reconstruction-only models it was compared with by Zimmerer et al. on each dataset. This model is also the basis of the WVALE method described in Section 5 for COVID-19 CT, which shows how directly this line of brain MRI work transfers to other modalities.

A second way is to fix the latent space directly. These models detect lesions by encoding a scan into a latent code, decoding it back into a healthy looking image, and identifying lesions from the difference between the original and reconstructed scans. Chen and Konukoglu [82] pointed out that nothing in the usual training makes this actually work, because a scan with a lesion can be encoded far from its healthy version, and the decoder then rebuilds the lesion instead of removing it, which leaves an empty residual. Their solution was to add a training term that pushes the latent code of an image with lesion towards the latent code of the same brain without the lesion, so that healthy and diseased inputs share the same region of the latent space and the reconstruction comes back healthy. In addition, the authors also applied their method to an adversarial autoencoder (AAE), which uses adversarial matching of its latent distribution instead of the VAE’s usual KL term. For training they used healthy T2-weighted scans from the Human Connectome Project [84], and for evaluation they used lesion masks from T2-weighted BraTS images [45]. The results showed an AUC of 0.897 for the VAE, 0.885 for the unconstrained AAE, and 0.923 for the constrained AAE; the last result does not belong to the VAE.

Two later works keep the context-encoding idea but change the engineering around it so that it works on real 3D data. Bengs et al. [85] start from the observation that most of these models are 2D and process the brain slice by slice, which ignores that lesions are three-dimensional. They move to a 3D VAE that takes the whole volume and add a spatial erasing task, a 3D version of the context-encoding idea of Zimmerer et al. [81] where sub-regions of the volume are masked and the network learns to fill them from their surroundings. On the same T1-weighted BraTS 2019 MRI [45] in Bengs et al.’s evaluation, the 3D VAE outperformed its 2D counterpart, and the spatial-erasing task added a further gain, raising the average Dice from 25.76% for the 2D VAE to 31.40% for the best 3D model with erasing. Chatterjee et al. [86] in their work mention that anomaly detection models often look good on artificial anomalies but fail on real clinical scans. To address this, their proposed approach StRegA keeps a compact ceVAE but adds preprocessing and postprocessing steps that make the anomaly map more stable on clinical data. Using T2-weighted BraTS tumor images [45], StRegA reached a Dice score of 0.642 against 0.522 for the best baseline in Chatterjee et al.’s evaluation; on artificially induced anomalies in a separate experiment, it reached 0.859 against 0.783 for the baseline. This shows how the processing around the VAE affected the results in their experiments.

The last group changes the latent representation itself. Pinaya et al. [87] proposed an unsupervised framework that combines a VQ-VAE with autoregressive transformers for brain-anomaly detection and segmentation, and the full pipeline is shown in Figure 9. The VQ-VAE first compresses each MRI into a discrete latent representation, which is then converted into a sequence and analysed by an ensemble of autoregressive transformers trained on largely healthy UK Biobank scans [88], whose architecture is detailed in Figure 10. Low-probability latent codes are treated as abnormal, replaced with more likely values, and decoded to produce a healed image. The final lesion map is obtained from the difference between the original and healed scans, filtered using the transformer’s resampling mask to reduce false positives caused by reconstruction errors. The method was evaluated on synthetic data and real FLAIR datasets containing brain tumors, multiple-sclerosis lesions, and white-matter hyperintensities. In the 3D experiments, it achieved Dice scores of 0.617 on BraTS [45], 0.368 on UK Biobank [88], and 0.133 on both MSLUB [89] and WMH [90], outperforming the compared dense VAE on the same test data in Pinaya et al.’s evaluation. However, these results used the best achievable threshold for each dataset, and performance decreased on external datasets; they should not be read as performance at a fixed deployment threshold.

Wijanarko et al. [91] proposed Tri-VAE, a triplet variational autoencoder for unsupervised brain-tumor detection from MRI. The model was trained only on healthy T2-weighted scans from 250 IXI subjects [92] and evaluated on 30 BraTS 2020 tumor cases [45]. During the training phase, three healthy 2D slices were selected as anchor, positive, and negative samples. For the negative slice, they added structured noise to simulate an abnormal region. The shared VAE was then trained with a triplet loss to distinguish healthy and corrupted representations while reconstructing the noisy slice as its original clean version. A semantic-guided gated skip module preserved spatial details in healthy regions while limiting the transfer of anomalous features to the decoder. At inference, lesions were identified from the residual between the pathological slice and its healthy-looking reconstruction. Using coarse noise on the same 30 preprocessed T2-weighted BraTS 2020 test scans, Tri-VAE achieved a Dice score of 0.606, an AUROC of 0.968, and an AUPRC of 0.462, outperforming the compared restoration-based VAE, DAE, AnoDDPM, f-AnoGAN, and ASC-Net methods in Wijanarko et al.’s Dice evaluation on those scans.

Overall, the reported results suggest that the reconstruction-based anomaly segmentation is still less precise than fully supervised segmentation. The Dice scores reported in these studies vary in a considerable way, but these values should be interpreted carefully because the studies use different pathologies, datasets, preprocessing pipelines, image resolutions, and thresholding strategies. These methods are also usually designed to identify abnormal regions without requiring lesion annotations, rather than to produce highly precise clinical contours. Their current usefulness may therefore be more in highlighting suspicious regions, supporting screening, or providing initial candidate masks than in replacing supervised segmentation when accurate boundary delineation is required. The large variation in performance across datasets also highlights the need for stronger external validation and better robustness to domain shift before these approaches can be considered reliable for clinical use.

### 6.5. Cardiac MRI Segmentation

Beyond brain imaging, VAEs are often used to learn realistic anatomical shapes rather than for anomaly detection or regularization. This is especially useful in cardiac MRI segmentation, where a model can obtain a high Dice score but still produce an unrealistic heart shape, such as a hole in the myocardium or a disconnected ventricle. Hence, segmentation performance should not be judged on overlap metrics alone, but also on whether the predicted anatomy is realistic. This follows the same idea as the shape-prior methods discussed for CT in Section 5.

Skandarani et al. [93] have proposed a hybrid VAE–GAN framework that generates synthetic cine-MRI images together with their segmentation masks. The VAE is first trained to learn the latent distribution of cardiac anatomical shapes, from which new plausible segmentation masks can be sampled. These masks are then provided to a SPADE-based GAN that generates realistic cardiac MR images that follow the corresponding anatomy. They evaluated their approach on the ACDC dataset [94] and the Sunnybrook Cardiac Data dataset [95]. The generated image–mask pairs were used to train a separate segmentation network, and the synthetic data improved the segmentation performance, particularly when combined with fine-tuning on real images. On the Sunnybrook dataset, for example, the Dice score increased from 0.776 when training on the original data to 0.874 when using the generated data followed by fine-tuning. This work represents Role A, where the VAE is used to generate anatomical shapes that support synthetic data augmentation for segmentation. Habijan et al. [96] proposed a 3D encoder–decoder architecture with VAE regularization for whole-heart segmentation. The encoder is based on feature-merge pre-activation residual blocks (FM-Pre-ResNet), while a VAE branch reconstructs the input image from the latent representation learned by the encoder. The reconstruction and the KL-divergence losses are used together with the segmentation loss to regularize the shared encoder and reduce overfitting. The VAE branch is used only during training, while the decoder produces the final whole-heart segmentation. They evaluated their approach on the MM-WHS dataset [97] using both cardiac CT and MRI images and their method has achieved an average Dice scores of 90.39% for CT and 89.50% for MRI. Hence, this work can be classified in Role B, where the VAE is integrated into the segmentation network as an auxiliary regularization mechanism. Cui et al. [98] have proposed a VAE-based unsupervised domain adaptation framework for cardiac MRI segmentation, with the aim of transferring the knowledge learned from annotated bSSFP images to LGE images where it is difficult to obtain manual annotations. Their approach introduces VAMCEI, which is a variational feature alignment module that maps the source and target images into latent distributions and uses the KL divergence between them to reduce the global difference between the two domains. In addition to this global alignment, prototype contrastive learning is used to bring closer the features that belong to the same anatomical structure across the two domains, while a domain discriminator provides an additional implicit alignment of the segmentation features. The authors further combined VAMCEI with CycleGAN to construct a multi-stage domain adaptation framework and evaluated it on the MS-CMRSeg 2019 [99], MyoPS 2020 [100], and MM-WHS 2017 [97] datasets. The multi-stage framework achieved an average Dice score of 85.96% on MS-CMRSeg and 81.20% on MyoPS. This work therefore represents Role B, where the VAE latent space is used to reduce the distribution difference between the source and target domains and improve segmentation under cross-modality domain shift.

Painchaud et al. [101] proposed a method that corrects cardiac segmentation masks when their shapes are not anatomically valid. A constrained VAE was trained on heart masks drawn by medical experts from the ACDC dataset to learn a latent space of valid cardiac shapes. A simple regression branch was added to organize this space according to the position of each MRI slice from the base to the apex, allowing nearby latent points to produce smooth changes in heart shape. To create a large set of reliable heart shapes, the authors sampled many points from the latent space and decoded them into segmentation masks. Each mask was checked using anatomical rules; if the mask has no holes, separated structures, or incorrect contact between regions, it is kept; otherwise, it is rejected. When a new segmentation contains an anatomical error, it is encoded into the latent space in which it is gradually shifted toward the nearest valid point until the decoded mask passed all the checks. The method was validated on short-axis cardiac MRI from the ACDC dataset [94] and on echocardiography from the CAMUS dataset [102], and it removed the anatomically impossible cases from the output of several segmentation networks without lowering their Dice. This result also shows an important limitation of evaluating cardiac segmentation with only overlap-based metrics. Two segmentations can reach similar Dice scores while being very different in their anatomical validity, for example through disconnected regions, holes, or incorrect relationships between the cardiac structures. For this reason, cardiac segmentation can benefit from complementary topology- and shape-aware evaluation, together with clinically relevant measurements derived from the predicted masks. The work of Painchaud et al. [101] is particularly relevant in this context, because it explicitly checks whether a predicted segmentation represents a plausible cardiac anatomy rather than relying only on the pixel-wise overlap. This is the MRI counterpart of the CT shape-prior work of Sandfort et al. [58], and it is a clear example of Role E, where the VAE is used as an anatomical shape prior to correct implausible segmentation masks.

A different use of the VAE in cardiac MRI is for representation learning rather than shape correction. Qiao et al. [103] proposed CMRVAE, a contrastive margin-restrained VAE for unsupervised domain adaptation, where a model trained on a labeled source domain has to segment images from a different and unlabeled target domain, such as another scanner or another acquisition sequence. Domain shift is a problem because the features of the two domains do not align, so a network trained on one performs poorly on the other. CMRVAE addresses this in the latent space. It applies patch-level contrastive learning on the VAE manifold and uses the labels of the source domain to guide it, so that patches of the same class are pulled together and patches of different classes are pushed apart across both domains. The result is a latent space that is separated by class and shared between the two domains, which is then used to segment images from the unlabeled target domain. Biffi et al. [104] proposed a conditional VAE to reconstruct a high-resolution 3D segmentation of the left ventricle from only three sparse 2D segmentations, corresponding to one short-axis and two long-axis views. The 2D masks are first encoded and used as conditions for the variational model, while the decoder learns to reconstruct the complete 3D shape of the left ventricle. The model was trained using cardiac MRI from 1912 healthy subjects and evaluated on 400 unseen cases. Using the three available views on the same 400 held-out cardiac MRI subjects, the proposed CVAE achieved a Dice score of 87.92±0.15%, compared with 82.60±0.23% for the TL-net baseline in Biffi et al.’s evaluation. An interesting property of the variational formulation is that different latent samples can also be decoded to form voxel-wise maps of the reconstructed cardiac shape. Biffi et al. called these confidence maps; they show variation among sampled masks rather than a validated measure of clinical confidence. This work therefore shows another use of VAEs as anatomical shape priors (Role E), where a complete 3D segmentation can be inferred from sparse segmentation information.

Overall, the studies in this section show that VAEs contribute to MRI segmentation in several ways. They can support supervised segmentation through latent representation integration and regularization (Role B), generate synthetic training samples for data augmentation (Role A), detect and localize abnormalities in brain MRI without manual labels (Role C), and learn anatomical shape information for segmentation correction and quality control in cardiac imaging (Role E). Table 6 summarizes these studies according to their role, VAE type, task, datasets, and main reported results.

## 7. VAEs for Retinal Vessel Segmentation

Retinal vessel segmentation plays an important role in the diagnosis of eye diseases such as diabetic retinopathy, since changes in vessel shape and density can reveal early signs of the disease. Outside brain and cardiac imaging, the retina is one of the main areas where VAEs are used for segmentation. The main challenge is the limited amount of annotated data and the large differences between datasets. Fundus images may be captured with different cameras, at different resolutions, or with another imaging method such as OCTA. As a result, a model that is trained on one dataset may not perform well on another. VAEs can generate synthetic samples to improve segmentation model performance. They are also often combined with GANs, which makes retinal imaging a useful example of the hybrid approaches discussed in Section 10.

Hu et al. [105] addressed domain shift in retinal vessel segmentation by training a model that could be applied to datasets not seen during training. Their method combines three components. First, a full-resolution variational autoencoder, called f-VAE that produces a latent image with the same width and height as the input. Rather than sampling latent vectors and decoding them into new fundus images, the authors trained three separate f-VAE models whose encoders generated latent images with different visual styles but similar vessel structures. These images were used as additional pseudo-domains for training. Second, each image was converted into a Hessian-based vector field that represents the local direction of the vessels. Since the tubular vessel shape remains similar across datasets and imaging types, this representation reduces the effect of changes in brightness and appearance. A vector field transformer then used this information at several scales to produce the final segmentation. The method was evaluated on four color fundus datasets, DRIVE [106], STARE [107], ARIA [108], and HRF [109], and two OCTA datasets, OCTA-500 [110] and ROSE [111]. When the model was trained on fundus images and then evaluated on datasets that were not used during training, it reached Dice scores of 0.706, 0.573, and 0.641 for the healthy, diabetic, and glaucoma groups in HRF, respectively. It also achieved 0.779 on OCTA-500 and 0.728 on ROSE, showing better Dice than a standard residual U-Net trained without domain-generalization techniques on the same HRF, OCTA-500, and ROSE target datasets in Hu et al.’s evaluation.

Radha and Karuna [112] also used a VAE to expand the available retinal training data. Their conditional GAN model translates vessel masks into fundus images and fundus images into vessel masks, producing image and mask pairs that can be used to train a separate segmentation model. Inside the generator, a variational autoencoder is used to predict the mean and log-variance of the latent distribution which helps the model to keep the main vessel structure while introducing changes in the generated images. For the experiments, the authors used DRIVE [106], STARE [107], and CHASE_DB [113] datasets. They selected 440 synthetic pairs of size 256×256, keeping samples with SSIM above 0.9 and PSNR above 30 dB. A simple U-Net trained on these synthetic images reached an accuracy of 0.996, an AUC of 0.990, and an IoU of 0.983 on the same 44 held-out retinal images, compared with 0.965, 0.986, and 0.702 when trained on the original data. Both versions used images resized to 256×256 and patching. However, this large improvement needs to be interpreted carefully. The increase in the IoU from 0.702 to 0.983 is unusually high for retinal vessel segmentation and it was obtained under the experimental setting used by Radha and Karuna [112]. The authors did not mention any independent external validation to verify whether the same improvement can be reproduced on data acquired under different conditions. Therefore, these results show the potential of synthetic data augmentation in improving the performance of models in segmentation, however, they should not be generalized without external validation.

Taken together, the works on retinal vessel segmentation show that VAEs are mainly used to address the shortage of annotated data and the variation between datasets. They can generate synthetic training samples or pseudo-domains for augmentation and domain generalization (Role A), or contribute directly to the segmentation network through latent representation integration and regularization, often in combination with a GAN (Role B). Table 7 summarizes these methods, together with the VAE variant, the task, the datasets used, and the reported results.

## 8. Multimodal and Missing-Modality Segmentation with VAEs

The previous sections considered each imaging modality separately, but many medical imaging tasks use several types of images together. For instance, in the MRI modality, in one examination, we can have different types, like T1, contrast-enhanced T1, T2, and FLAIR sequences, with each one showing different tissue features. Combining these sequences can improve segmentation because they provide complementary information. However, this also introduces two main challenges. First, the model must combine the information from the different sequences effectively. Second, some sequences may be missing because of limited scanning time, cost, patient movement, or differences in hospital protocols, meaning that a model that depends on having every sequence available may therefore perform poorly in real clinical settings.

VAEs are considered to be useful particularly in this setting because they can represent information from different modalities within a common latent space. Each MRI sequence can be processed by its own encoder, while the resulting representations are aligned so that the shared latent space captures the underlying anatomy rather than the appearance of a specific sequence. From this representation, the model can reconstruct an unavailable modality and produce the segmentation mask. Hence, missing modalities can be handled as information that must be inferred from the available sequences, rather than requiring a separate model for every possible combination of inputs. This approach is closely related to multimodal and missing-modality segmentation and builds on the multimodal VAE frameworks introduced in Section 2, including the product-of-experts MVAE [22], the mixture-of-experts MMVAE [23], and the mixture-of-products-of-experts MoPoE-VAE [24].

This section is divided into three parts. We first introduce the multimodal VAE principles that allow information from different modalities to be represented in a shared latent space. We review then their use in brain tumor segmentation with missing MRI sequences, which stays the most widely studied application. Finally, we examine more recent approaches that extend these principles to different imaging modalities and patterns of missing data.

### 8.1. Multimodal VAE Foundations

The multimodal VAE architectures that are introduced in Section 2 provide the main foundation for handling incomplete multimodal inputs. In these models, each modality is processed by a separate encoder, but all encoders represent their information in a common latent space. Their modality-specific posterior distributions can then be combined using a product of experts, as in MVAE [22], a mixture of experts, as in MMVAE [23], or a mixture of products of experts, as in MoPoE-VAE [24]. A key advantage of these formulations is that they do not require the same modalities to be available for every sample. When one modality is missing, its encoder then is eliminated and the available modalities are still used to infer a valid latent representation. The corresponding decoders can then reconstruct the unavailable modality or support another downstream task, such as segmentation. Although these models were developed initially for general multimodal settings, including images paired with attributes or other data types, later medical applications adapted the same principle to MRI, where the modalities correspond to different sequences and the shared representation is used to segment the tumor despite incomplete inputs.

### 8.2. Missing-Modality Brain Tumor Segmentation

The most widely studied application of these ideas is brain tumor segmentation using the four MRI sequences of the BraTS challenge [45], particularly when one or more sequences are unavailable. An important approach was the one proposed in HeMIS [115], which processes each sequence through a separate convolutional pathway and combines the resulting feature maps by calculating their mean and variance across the available modalities. These statistics can be computed from any subset of sequences, allowing the same model to operate under different missing-modality conditions. Although HeMIS generally maintains a gradual decline in performance as sequences are removed, its fusion is limited to fixed summary statistics. It does not explicitly learn a probabilistic joint distribution of the modalities or reconstruct the missing sequences, which later variational approaches attempt to address.

U-HVED [116] (Figure 11) reformulated missing-modality brain tumor segmentation within a variational framework. The model combines a 3D U-Net with the multimodal VAE described in Section 8.1. Each of the four MRI sequences is processed by a separate encoder that predicts a Gaussian distribution, and the distributions of the available sequences are combined through a product of experts to form a shared latent representation. This allows the same network to accept any non-empty subset of the four sequences. From the shared representation, four modality-specific decoders reconstruct the MRI sequences, while an additional decoder predicts the tumor regions, giving n encoders and n + 1 decoders for n modalities. During training, complete BraTS cases are presented with randomly removed input sequences, while reconstruction is evaluated using all available sequences. This encourages the latent representation to remain consistent in the different modality subsets and to preserve information useful for both modality completion and segmentation. The authors evaluated their approach on BraTS 2018 [45] across all fifteen nonempty modality combinations, and the results showed that their model U-HVED achieved average Dice scores of 0.801 for the whole tumor, 0.640 for the tumor core, and 0.500 for the enhancing tumor, compared with 0.507, 0.287, and 0.274 for HeMIS evaluated on the same modality combinations in Dorent et al.’s study. Its results were also close to those of fifteen separately trained subset-specific networks, which obtained 0.816, 0.662, and 0.527, respectively.

U-HVED combines the available MRI sequences of each patient through a product of experts but retains the standard Gaussian prior of a conventional VAE. Hamghalam et al. [117] argued that this assumption does not explicitly capture the relationships that exist across patients or between MRI sub-modalities. Their MGP-VAE therefore replaces the independent Gaussian prior with a Gaussian process prior whose covariance includes both a patient kernel and a modality kernel. In this framework, the latent code of a missing sequence is predicted using the encoded representations of the available samples together with the learned patient and modality correlations, and the decoder then reconstructs the missing MRI volume. The available and imputed sequences are subsequently concatenated and passed to a separate 3D U-Net for tumor segmentation. Using BraTS 2019 [45], the authors evaluated their approach with one, two, or three of the four sequences missing, and MGP-VAE achieved median Dice scores of 0.835 for the whole tumor, 0.656 for the tumor core, and 0.507 for the enhancing tumor, compared with 0.821, 0.634, and 0.494 for HVED under the same missing-modality settings in Hamghalam et al.’s evaluation. This is separate from Dorent et al.’s BraTS 2018 results. These results suggest that modeling relationships across both patients and MRI sequences can improve modality completion and the segmentation that follows.

A different way to improve U-HVED is to make the information used for segmentation focus more strongly on the lesion. Jeong et al. [118] proposed RA-HVED, a region-of-interest attentive extension of the hetero-modal variational encoder–decoder. It retains U-HVED’s multimodal VAE and product-of-experts fusion but adds two main mechanisms related to segmentation. First, its ROI-attentive skip connections use the segmentation features to weight the encoder features before they are passed to the decoder. This emphasizes information from likely tumor regions while reducing the influence of less relevant background structures. Second, a joint discriminator receives the ROI-attentive reconstructed images together with their segmentation probability maps. The resulting adversarial loss encourages modality completion from incomplete inputs to preserve lesion-related information rather than focusing only on the overall appearance of the reconstructed MRI. The authors evaluated their approach on BraTS 2018 [45] across all 15 nonempty combinations of available sequences, and the results showed that RA-HVED achieved average Dice scores of 0.842, 0.756, and 0.549 for the whole tumor, tumor core, and enhancing tumor, compared with 0.811, 0.734, and 0.510 for U-HVED under the same protocol. It also improved the average Dice on the ISLES 2015 stroke dataset [83] from 0.429 to 0.483 in Jeong et al.’s comparison. These U-HVED results differ from the previous ones because Jeong et al. re-evaluated U-HVED under their own implementation and protocol rather than using the scores of Dorent et al. [116].

The most recent work in this line retains the hetero-modal VAE framework while adding a more recent sequence-processing module. Zhu et al. [119] proposed XLSTM-HVED, which continues to combine the available MRI sequences through a multimodal VAE and a product of Gaussian experts, allowing the distribution of a missing sequence to be excluded from the fusion. Rather than replacing all convolutional blocks, the model introduces a Vision-xLSTM attention module at the deepest encoder level, where the 3D feature volume is reshaped into a sequence of spatial tokens to capture long-range dependencies. The authors also proposed a self-attention variational encoder that applies spatial attention to the modality features before fusion, and a squeeze-fusion-excitation cross-awareness module that exchanges channel and spatial information between the reconstruction and segmentation decoders. This work was evaluated on BraTS 2024, and across all 15 nonempty combinations of the four sequences, XLSTM-HVED reached average Dice scores of 74.53% for the whole tumor, 58.19% for the tumor core, and 53.83% for the enhancing tumor, compared with 68.82%, 47.82%, and 39.77% for RA-HVED under the same random 80/20 train/test split and U-HVED backbone in Zhu et al.’s study. These scores are separate from Jeong et al.’s BraTS 2018 results. These results show that the multimodal VAE foundation of U-HVED can still benefit from stronger long-range feature modeling and closer interaction between reconstruction and segmentation, particularly for the tumor core and enhancing tumor.

### 8.3. Cross-Modality Synthesis as a Related Application

The previous works focused mainly on the four MRI sequences used in BraTS. Dorent et al. [120] in their approach have examined whether the same probabilistic fusion principle could be extended to more distinct imaging modalities through MHVAE, which was proposed for synthesis between preoperative T2 MRI and intraoperative ultrasound. This work is related to the multimodal segmentation setting, but it does not perform segmentation itself. Their approach MHVAE uses a hierarchy of latent variables rather than relying on a single compact latent variable. In this hierarchy, the coarser levels capture global anatomical structure, while the finer levels preserve local details and image texture. At every level, the posterior estimates produced from the available modalities are combined through a product of experts, allowing one model to synthesize ultrasound from MRI, synthesize MRI from ultrasound, or reconstruct both when both are available. No segmentation results were reported. Nevertheless, the study shows that multimodal VAE fusion can be extended beyond related MRI sequences to modalities with very different appearances and acquisition processes.

Taken together, these studies show that multimodal VAEs can combine information from several available modalities in a shared latent space and continue performing segmentation when some inputs are missing. However, the evidence reviewed here is still concentrated on brain MRI and missing-sequence tumor segmentation, so it remains unclear whether the same approach can generalize effectively to other organs and more distinct imaging modalities. Another limitation is that the reconstruction branch used to organize the latent space can produce smooth or blurry images, which may reduce the quality of the completed modalities. Recent studies also combine the variational framework with other components such as attention mechanisms, transformers, or xLSTM modules. These examples suggest that VAEs can serve as parts of larger hybrid architectures. These challenges are discussed further in Section 10.

Table 8 summarizes the multimodal works reviewed in this section. The missing-modality segmentation methods correspond to Role D and are mainly based on hetero-modal VAE frameworks, while MHVAE is included separately as a related cross-modality synthesis application.

## 9. Datasets and Evaluation

The works reviewed in this survey are evaluated on a range of public datasets and with a shared set of metrics. This section collects the datasets used across the different modalities and recalls the measures that appear in the reported results.

### 9.1. Benchmark Datasets for Segmentation

The reviewed studies used several public datasets that appear repeatedly across imaging areas. We first introduce these datasets by modality and application, and then provide a summary of them in Table 9. Brain MRI datasets, especially those from the BraTS challenge, are used most frequently. Their popularity is largely due to the availability of relatively large, annotated, multi-sequence collections, which has made brain tumor segmentation the main application of VAEs in medical image segmentation.

**Brain MRI.**

- **BraTS** [45]: The Brain Tumor Segmentation challenge, a multi-institutional collection of pre-operative glioma scans with four co-registered sequences (T1, contrast-enhanced T1, T2, and FLAIR) and expert annotations of the whole tumor, the tumor core, and the enhancing tumor. It is released in yearly editions of growing size and is the main benchmark of this review.
- **ISLES 2015** [83]: The Ischemic Stroke Lesion Segmentation challenge, with multi-spectral MRI and manual annotations of stroke lesions.
- **HCP** [84]: The Human Connectome Project, a large set of high-quality MRI from healthy young adults, used as normal data by the unsupervised anomaly methods.
- **UK Biobank** [88]: A very large population study that includes brain MRI of tens of thousands of healthy subjects, also used as normative data.
- **MSLUB** [89]: An MRI dataset of multiple sclerosis patients with lesion segmentations obtained from a multi-rater consensus.
- **WMH** [90]: The White Matter Hyperintensity segmentation challenge, with FLAIR and T1 scans from several scanners and manual annotations of white-matter hyperintensities.
- **IXI** [92]: A collection of MRI from nearly six hundred healthy subjects acquired at several hospitals, used as normal data.
- **OASIS** [78]: An open-access collection of structural brain MRI scans from healthy and cognitively impaired subjects, used in the reviewed few-shot segmentation study for anatomical segmentation.
- **QUBIQ** [43]: A benchmark designed for uncertainty quantification in biomedical image segmentation, where several expert annotations are available for the same images. The reviewed works use its brain-growth MRI task as well as its prostate and abdominal segmentation tasks.

**Cardiac.**

- **ACDC** [94]: The Automated Cardiac Diagnosis Challenge, with cine MRI from 150 patients across five diagnostic groups and annotations of the left ventricle, the right ventricle, and the myocardium.
- **CAMUS** [102]: A two-dimensional echocardiography dataset from 500 patients with annotations of the cardiac structures.
- **Sunnybrook** [95]: A cardiac MRI dataset introduced for the left-ventricle segmentation challenge and containing short-axis cine-MRI images with manual ventricular annotations.
- **MM-WHS** [97]: The Multi-Modality Whole Heart Segmentation challenge, containing cardiac CT and MRI images with annotations of the main cardiac structures.
- **MS-CMRSeg** [99]: A multi-sequence cardiac MRI benchmark including bSSFP, T2-weighted and LGE images, used for cross-sequence cardiac segmentation and domain adaptation.
- **MyoPS** [100]: A cardiac MRI benchmark combining multiple MRI sequences for the segmentation of myocardial structures and pathological regions.

**Abdominal and other CT.**

- **NIH Pancreas-CT** [62]: An abdominal CT dataset with manual pancreas annotations, commonly used for pancreas segmentation.
- **Medical Segmentation Decathlon (MSD)** [63]: A multi-task medical segmentation benchmark covering several anatomical structures and imaging modalities. The pancreas CT task is used by one of the reviewed domain-adaptation studies.
- **Synapse/BCV** [64]: A contrast-enhanced abdominal CT dataset with annotations of multiple abdominal organs, commonly used for multi-organ segmentation.
- **CHAOS** [60]: A combined CT and MRI abdominal organ segmentation benchmark containing annotations of structures such as the liver, kidneys and spleen.
- **KiTS19** [70]: A kidney tumor CT benchmark containing kidney and tumor annotations for segmentation.
- **VerSe** [79]: A CT benchmark for vertebra localization, labeling and segmentation across scans acquired with different CT systems.

**Lung and chest CT.**

- **LIDC-IDRI** [40]: The Lung Image Database Consortium collection, with more than a thousand thoracic CT scans and lung nodule annotations from several radiologists.
- **LNDb** [53]: A lung nodule CT database with nodule annotations.
- **NSCLC radiogenomics** [54]: CT scans of non-small-cell lung cancer patients with tumor segmentations and matched genomic data.
- **NLST** [56]: The National Lung Screening Trial, a large low-dose CT lung cancer screening dataset.

**PET/CT.**

- **HECKTOR** [74]: The Head and Neck Tumor segmentation challenge, with FDG-PET/CT scans and annotations of the primary tumors.

**Mammography.**

- **INbreast** [37]: A full-field digital mammography dataset containing annotated breast abnormalities, including mass lesions.
- **CBIS-DDSM** [38]: A curated version of the DDSM mammography collection with standardized images and annotations for breast lesions.

**Retinal.**

- **DRIVE** [106]: A set of 40 color fundus images with manual vessel annotations, the most classical retinal benchmark.
- **STARE** [107]: A set of 20 color fundus images with vessel annotations from two experts.
- **ARIA** [108]: Color fundus images with vessel annotations, including healthy, diabetic, and age-related macular degeneration subjects.
- **HRF** [109]: 45 high-resolution color fundus images from healthy, diabetic retinopathy, and glaucomatous subjects, with vessel gold standards.
- **OCTA-500** [110]: An OCT angiography dataset of 500 subjects with vessel and structure annotations.
- **ROSE** [111]: An OCT angiography dataset with pixel-level and centerline-level vessel annotations.
- **CHASE_DB** [113]: A set of 28 color fundus images from schoolchildren with vessel annotations from two observers.

### 9.2. Evaluation Metrics for VAE-Based Segmentation Pipelines

Most of the reviewed works measure the overlap between the predicted region *P* and the reference region *G* with the Dice similarity coefficient (DSC),

(13) $$\mathrm{DSC}=\frac{2\;|P\cap G|}{\left|P|+|G\right|},$$

and, equivalently, with the Jaccard index or intersection over union (IoU),

(14) $$\mathrm{IoU}=\frac{\left|P\cap G\right|}{\left|P\cup G\right|}.$$

Both range from 0 to 1 and increase with better overlap. When the results are reported at the pixel level, in particular for retinal vessels, the sensitivity (recall), specificity, and accuracy are used, defined from the true and false positives and negatives (TP, FP, TN, FN) as

(15) $$\mathrm{Se}=\frac{TP}{TP+FN}\;\mathrm{Sp}=\frac{TN}{TN+FP}\;\mathrm{Acc}=\frac{TP+TN}{TP+TN+FP+FN}$$

Unsupervised anomaly detection methods usually produce a continuous anomaly score or anomaly map rather than a binary segmentation. Their performance is therefore commonly assessed using the area under the receiver operating characteristic curve (AUROC) and the area under the precision–recall curve (AUPRC), which evaluate performance across different decision thresholds. Segmentation boundaries can also be evaluated using the Hausdorff distance, particularly its 95th percentile form, HD95.

Methods that use VAEs for image generation or modality completion generally report image quality metrics rather than segmentation overlap. The Fréchet inception distance (FID) compares the feature distributions of generated and real images, with lower values indicating greater similarity. The peak signal-to-noise ratio (PSNR) measures pixel-level reconstruction fidelity, while the structural similarity index (SSIM) evaluates similarity in image structure, contrast, and local intensity patterns.

Despite the fact that these metrics are used widely, none of them actually provides a complete picture of segmentation quality. Dice and IoU metrics measure the overlap between the predicted and reference regions, but they may hide important boundary errors. This is usually faced when the lesions are small or the anatomical structures are thin, where a small change in the boundary can have a large effect on the clinical interpretation. The pixel accuracy can also be misleading when, for example, the tumor covers only a small part of the observation, since correctly classifying the large background region can give a high accuracy even when the tumor itself is poorly segmented. Boundary-based metrics give complementary information. The Hausdorff distance measures the largest distance between the predicted and reference boundaries, but it is very sensitive to isolated outliers. HD95 reduces this sensitivity by taking the 95th percentile of the boundary distances instead of the maximum, and it is therefore usually more stable. For anomaly detection, AUROC summarizes the separation between normal and abnormal predictions across different thresholds, but it can stay relatively high when the dataset is strongly imbalanced. AUPRC is more informative in this case because it focuses directly on the precision and recall of the abnormal class. Finally, PSNR and SSIM describe the pixel-level and the structural similarity between the generated and the real images, but high values do not mean that the generated images are anatomically or clinically realistic. For this reason, these metrics should be interpreted together with the task, the dataset, and the evaluation protocol, and not as standalone measures of the model quality.

## 10. Challenges and Future Directions

Across the modalities reviewed in this survey, VAEs are used in the five roles introduced in Section 3: generative augmentation, latent representation integration and regularization, reconstruction-based anomaly localization, multimodal latent fusion and missing-modality segmentation, and anatomical shape priors and segmentation quality control. Looking at the works together, several limitations and future research directions can be identified.

### 10.1. Overcoming the Blurriness of VAEs with Hybrid Architectures

The most consistent limitation of the VAE, visible in almost every section of this review, is that its reconstructions are blurry. This comes from the way the model is trained: the latent variable is regularized towards a Gaussian prior, and the output is optimized with a pixel-wise likelihood that averages over all the plausible reconstructions instead of committing to a sharp one. For segmentation this blurriness has direct consequences, since the boundaries of the predicted regions are soft, the synthetic images used for augmentation lack fine detail, and the anomaly maps obtained from the reconstruction error are noisy.

One strategy to reduce this blurriness is to combine VAEs with generative adversarial networks (GANs). In this approach, the adversarial loss encourages the reconstructed images to be more realistic and less blurry. This approach is used in the discriminative Hamiltonian VAE for tumor augmentation [71], in the retinal vessel models VGAN [114] and the latent-space autoencoder GAN of Radha and Karuna [112], and in the AnoVAEGAN method evaluated by Baur et al. [80]. Another direction in this hybrid approach, in which VAEs are combined with other generative models, is to use attention-based models, like transformers or other methods that can capture wider spatial relationships instead of GANs. Citing some examples of this approach, Lyu and Shu [67] have proposed a two-stage cascade with attention gates, Pinaya et al. [87] have proposed VQ-VAE with autoregressive transformers, and the Vision xLSTM module used in XLSTM-HVED [119].

From these studies we can identify a common pattern. In the hybrid architectures, usually the VAE provides a structured latent space and helps the model deal with missing or uncertain inputs, while the other model improves image quality or captures wider spatial information. In some of the studies reviewed here, methods performed better than the baselines tested in those papers when the VAE was combined with another model, which suggests that VAEs can be useful as components within larger hybrid architectures in these examples. The evidence for this pattern is limited to the studies reviewed here.

### 10.2. Training Pathologies and the Quality of the Latent Space

The usefulness of a VAE for segmentation depends strongly on how well its latent space is learned. Two main problems can affect this. The first is the balance between the reconstruction loss and the KL divergence. If the KL term is too weak, the model can reconstruct images well but the latent space becomes poorly organized. If it is too strong, as in a β-VAE, the latent space becomes more regular but the reconstructions may become smoother and lose detail. For this reason, the balance between the two terms usually needs to be adjusted for each task.

A second problem is posterior collapse. This happens when the decoder becomes strong enough to reconstruct the input without using much information from the latent code. As a result, different inputs can be mapped to very similar latent representations, which makes the latent space less useful. This is particularly problematic for segmentation and anomaly detection, where the latent code should preserve meaningful anatomical information. Some reviewed methods reduce this risk indirectly. Pinaya et al. [87], for example, use a discrete latent codebook, while ceVAE [81] adds a context encoding task that encourages the latent representation to capture the global structure of the image. Learning latent spaces that remain informative, well organized, and useful for downstream tasks is therefore still an important challenge.

### 10.3. Data, Evaluation, and Generalization

In Table 9, we see that the majority of reviewed studies are evaluated on brain MRI, using the BraTS challenge [45], while retinal vessel datasets account for much of the remaining work. Other organs and imaging types, such as abdominal imaging, prostate imaging, and histopathology, are rarely studied. This makes it difficult to know whether the same behavior would be observed outside these well-established benchmarks. Dataset size is another limitation. Many studies are based on only a few dozen or a few hundred images. This partly explains why VAEs are often used for data augmentation, but it also means that small differences between methods may not always reflect a real improvement.

Generalization across scanners and hospitals is another important issue. Baur et al. [80] showed that the reconstruction in anomaly detection methods can be sensitive to domain shift, meaning that changes in the scanner or acquisition protocol may increase reconstruction error even in healthy tissue. This makes it harder to distinguish normal variations from real abnormalities and limits the reliability of reconstruction error as an anomaly signal. Hu et al. [105] directly addressed this problem for retinal vessel segmentation, but only a small number of works in this review study domain generalization explicitly.

Another important issue concerns the use of synthetic data. When a VAE generates images and masks for augmentation, it may introduce unrealistic anatomical structures or details that do not correspond to real pathology. Most studies mainly report the improvement obtained in the final segmentation task and sometimes use measures such as SSIM or PSNR to assess the generated images [112]. However, these measures do not confirm that the synthetic samples are clinically correct. Hence, a more thorough evaluation of clinical validity is still needed before generated medical images can be used with confidence in practice.

Beyond VAE-based approaches, recent works in computational imaging also show that the quality of the acquired and reconstructed images can have an important effect on the analysis that is performed afterwards. Pang et al. [122] reviewed recent developments in deep-tissue optical imaging and discussed different problems such as light scattering, absorption, and image distortion, as well as the use of computational and AI-based methods to improve image reconstruction. Zhu et al. [123] proposed a self-supervised denoising method for super-resolution fluorescence microscopy that reduces background artifacts and improves the quality of the reconstructed structures, which can also be useful for later tasks such as segmentation. In another work, Qian et al. [124] proposed an ensemble deep learning method for single-shot structured illumination microscopy to reconstruct super-resolved images while reducing the effect of noise and reconstruction artifacts. These works are not directly based on VAEs and do not study medical image segmentation itself, but they show that improving image quality before the segmentation step can be important for obtaining more reliable results.

### 10.4. Uncertainty and Ambiguity in Segmentation

One useful property of conditional VAE segmentation models is their ability to represent variation between plausible masks. A VAE learns a distribution instead of mapping an image to a single latent code. When the model is trained to predict masks conditioned on an image, sampling different codes can produce several plausible segmentations for that image. This is particularly relevant in medical imaging, where the exact boundary of a lesion is not always clear and different experts may produce slightly different annotations. However, uncertainty in segmentation can come from different sources. Aleatoric uncertainty comes from the ambiguity that already exists in the data, such as unclear lesion boundaries or differences between expert annotations. Epistemic uncertainty is related instead to the model itself and can result from limited training data or uncertainty in the learned parameters. Predictive uncertainty represents the overall uncertainty of the final prediction and can include both of these sources. Sampling latent codes reflects the variation in masks learned by a conditional segmentation model. It does not by itself measure uncertainty in the model parameters or show that the predicted probabilities are calibrated. The Probabilistic U-Net [39], for example, combines a U-Net with a conditional latent variable that can be sampled several times for the same input image. Each sample can produce a different plausible segmentation, allowing the model to represent cases where more than one annotation can be considered acceptable.

Baumgartner et al. [41] extend this idea with PHiSeg, where several latent variables are introduced at different spatial resolutions. Instead of representing the variability using only one latent vector, the model represents variation at several spatial resolutions, including both large structural differences and smaller local variations between plausible segmentations. These approaches show how the probabilistic latent space of a VAE can be directly used to model multiple segmentation hypotheses rather than simply returning one deterministic mask. Most of the works reviewed here, however, mainly use the VAE for representation learning, reconstruction, or data generation, and still return one final segmentation. As a result, many of these studies do not assess uncertainty in the predicted masks. Even when multiple masks are generated, their variability should be checked against expert disagreement and prediction errors before it is used to indicate reliability. Better ways of estimating and presenting predictive uncertainty could therefore be valuable, especially if they can show clinicians which parts of a prediction are less reliable.

### 10.5. From Benchmark Performance to Clinical Use

Although several VAE-based segmentation approaches have achieved promising results, most have only been evaluated retrospectively on benchmark datasets. Strong performance on these datasets does not guarantee similar results under real clinical conditions. Images acquired across hospitals may differ because of variations in scanners, acquisition protocols and patient populations. External and prospective validation is therefore needed to determine whether these models can maintain their performance beyond the datasets on which they were originally evaluated.

Segmentation metrics alone are also insufficient to determine whether a model is clinically useful. Reader studies involving radiologists or other clinicians can help assess whether the predicted segmentations improve clinical interpretation, reduce annotation time or support treatment planning. Variability between annotators should also be considered, as different experts may define different boundaries for the same lesion or anatomical structure. In these situations, relying on a single annotation as the ground truth may not fully capture the uncertainty associated with the segmentation task.

The failure cases of these models also require closer investigation. A model may achieve a high average Dice score while still performing poorly on small lesions, unusual anatomies, low-quality images or cases that differ from the training distribution. Understanding when and why these failures occur is therefore essential before considering clinical deployment. Regulatory and safety requirements must also be addressed when such systems are intended to support clinical decisions. Overall, moving from benchmark performance to clinical use requires more than strong segmentation results. It also requires external validation, prospective studies, clinical evaluation and a careful analysis of model failures.

### 10.6. VAEs in the Era of Diffusion and Foundation Models

Diffusion models have recently become widely used in medical imaging. Compared with standard VAEs, they can generate sharper and more detailed images because the generation is performed through several denoising steps instead of a single decoding operation. However, this does not necessarily mean that they are always better than VAEs. Their performance depends on the task, the dataset and the evaluation protocol. Another limitation is the computational cost, since diffusion models require several denoising steps during inference, which can become expensive for large 3D medical images. VAEs remain simpler and faster, especially when the objective is to learn a compact latent representation or perform rapid inference. A direct comparison between VAE- and diffusion-based anomaly segmentation can be found in the work of Wijanarko et al. [91]. Under the same experimental setting on 30 T2-weighted BraTS 2020 test scans, they compared their Tri-VAE with AnoDDPM [125] and a restoration-based VAE in Wijanarko et al.’s evaluation [91]. AnoDDPM achieved a Dice score of 0.236, while the restoration-based VAE reached 0.349. Their proposed Tri-VAE achieved a Dice score of 0.606 when coarse noise was used. These results show that diffusion models do not necessarily provide better anomaly segmentation than VAE-based approaches, and that the obtained performance also depends on how the latent space and the reconstruction process are designed.

VAEs have not disappeared, however; in some of the approaches discussed here, they serve as an internal component. Latent diffusion models [126], for example, use a VAE encoder to compress the input image into a lower-dimensional latent representation. The diffusion process is then performed in this compressed space instead of directly on the original image. After the denoising process, the VAE decoder maps the latent representation back to the image space. In this case, the VAE is not used as the main generative model, but as a representation module that reduces the dimensionality of the data and makes the diffusion process less computationally expensive.

Another important development is the rise of foundation models such as the Segment Anything Model [127] and its medical adaptations [128]. These models are segmentation models that aim to provide segmentation capabilities across different structures and tasks, usually through large-scale pretraining and prompt-based segmentation. Hence, their relation with VAEs should be considered as complementary rather than evolutionary. Foundation models do not generally inherit the probabilistic latent representation, KL regularization, or sampling mechanism of VAEs. Instead, VAEs can provide other useful properties, such as generative augmentation and the ability to handle missing modalities through a shared latent representation. Foundation models initially trained on natural images can also require additional adaptation to medical imaging because of the differences in image appearance, anatomical structures, and the three-dimensional nature of many medical scans.

These examples suggest that VAEs can continue to have a relevant role in medical image segmentation, including as components within larger architectures. In those architectures, the VAE can provide a compact latent representation, regularize the segmentation network, support missing modalities, and, when trained to generate masks, represent variation between plausible segmentations. Multimodal VAEs are particularly interesting in this context because they can combine different inputs while continuing to operate when some modalities are unavailable. These properties suggest that VAEs can remain useful alongside foundation models in the applications discussed here.

## 11. Conclusions

This survey reviewed the use of variational autoencoders for medical image segmentation, organized around five roles that a VAE can play: generating synthetic data for augmentation, integrating variational representations into segmentation networks for representation learning and regularization, localizing pathology through reconstruction-based anomaly detection, combining multiple modalities in a shared latent space for missing-modality segmentation, and learning anatomical shape priors for segmentation correction and quality control. Across different medical imaging modalities, the same roles appear in different clinical problems, showing that the function of the VAE often depends more on the available data and the objective of the model than on the anatomy itself.

A number of recurring patterns can also be observed in the studies reviewed here. Several methods use VAEs as parts of larger systems. In these examples, their structured and probabilistic latent representations can support representation learning and the handling of missing modalities. Conditional segmentation models can also generate different plausible masks, but this alone does not show that their uncertainty estimates are calibrated. However, VAEs can still suffer from smooth and blurry reconstructions. For this reason, several recent approaches combine the VAE with other models, such as GANs, transformers, or more recently diffusion models, in order to improve image detail, capture wider spatial relationships, or complement the limitations of the variational representation.

However, the current literature is still concentrated on a limited number of medical imaging applications. Brain MRI, particularly the BraTS challenge, and a relatively small number of benchmark datasets are used in a large part of the reviewed studies, while other organs, modalities, and clinical settings remain less explored. The problem of generalization across datasets and institutions also still receives limited attention. Therefore, the five roles proposed in this review represent the main VAE applications identified in the reviewed literature and should not be considered as an exhaustive classification of all the possible uses of VAEs in medical image segmentation. More studies on different datasets, modalities, and clinical settings are still needed to evaluate how well these approaches can generalize beyond the currently studied applications.

Multimodal applications of VAEs can also be considered as an important direction for future work. A shared latent representation can combine information from different modalities while still allowing the model to operate when some inputs are missing. In this context, VAEs can continue to be useful as components of larger segmentation frameworks, where they can provide compact and structured latent representations, support missing-modality learning, and generate plausible mask alternatives when trained for that purpose. Their role can therefore remain complementary to other architectures such as transformers, foundation models, and diffusion models.

## Figures and Tables

**Figure 1:**
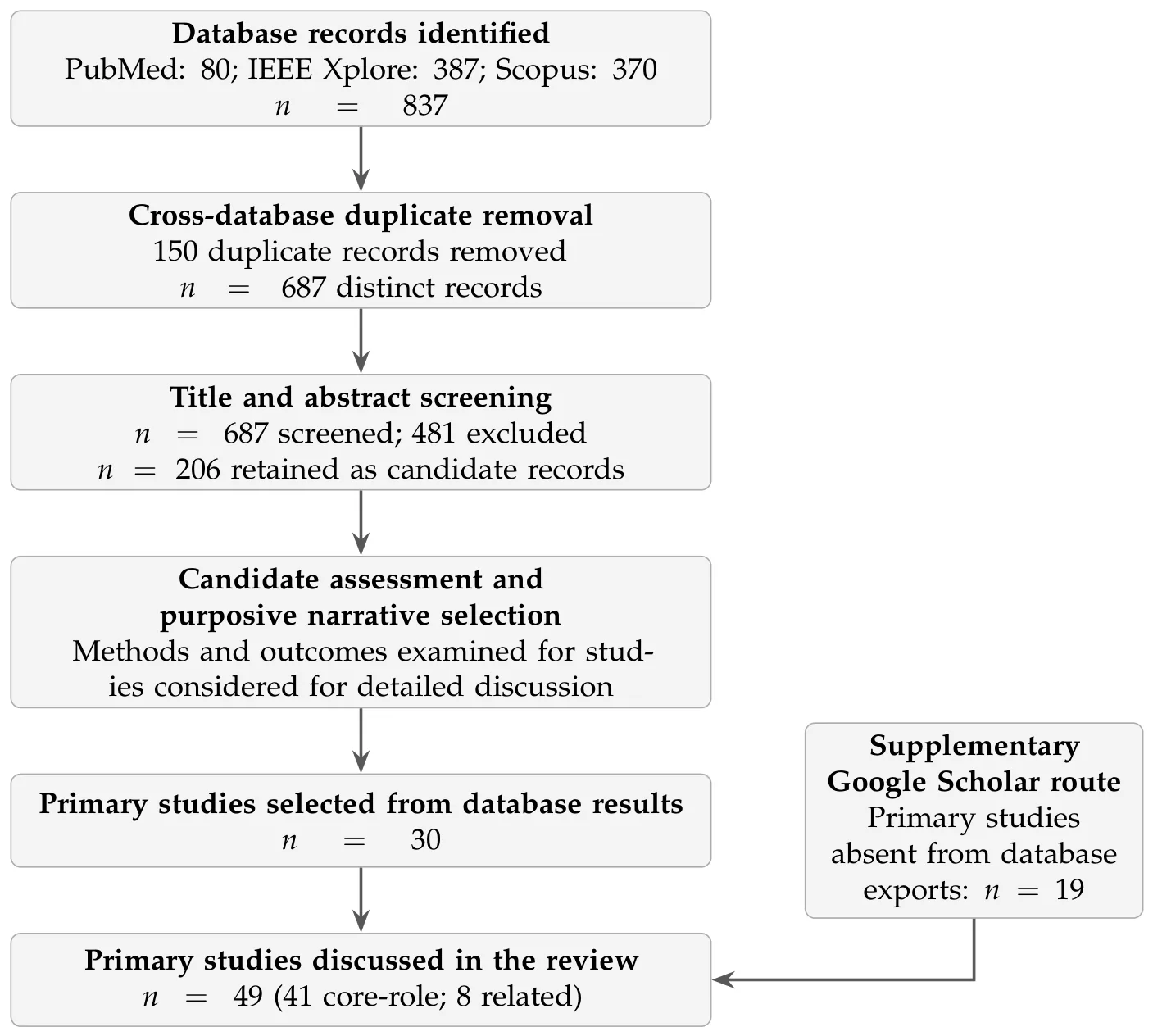
Identification and selection of primary studies for the review.

**Figure 2:**
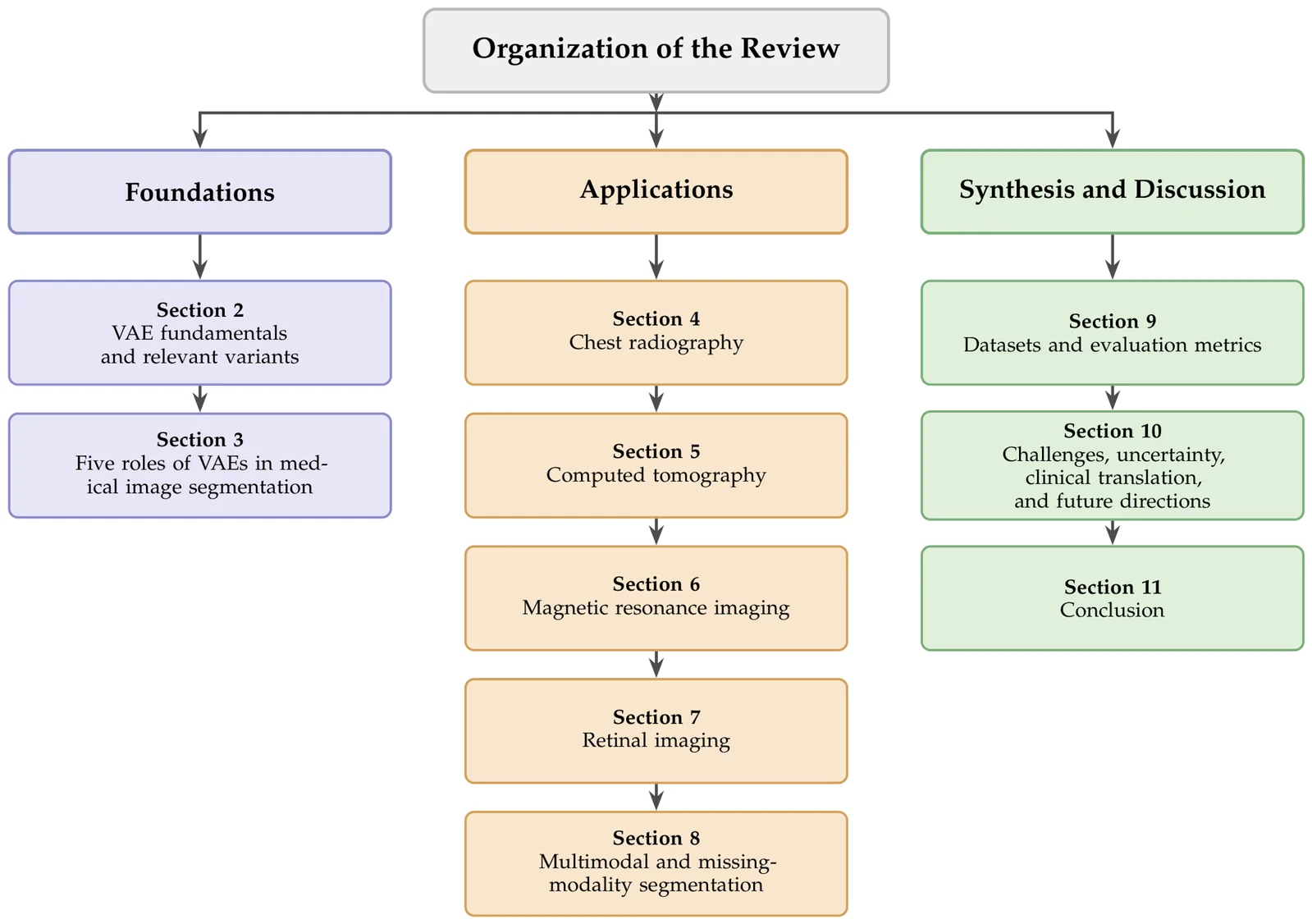
Organization of the present review. The manuscript first introduces the VAE fundamentals and the proposed five-role taxonomy (Foundations, Section 2 and Section 3). It then reviews VAE-based methods across five medical imaging domains (Applications, Section 4, Section 5, Section 6, Section 7 and Section 8). Finally, it summarizes the datasets and evaluation metrics and discusses the main challenges, clinical translation, and future directions before concluding the review (Synthesis and Discussion, Section 9, Section 10 and Section 11).

**Figure 3:**
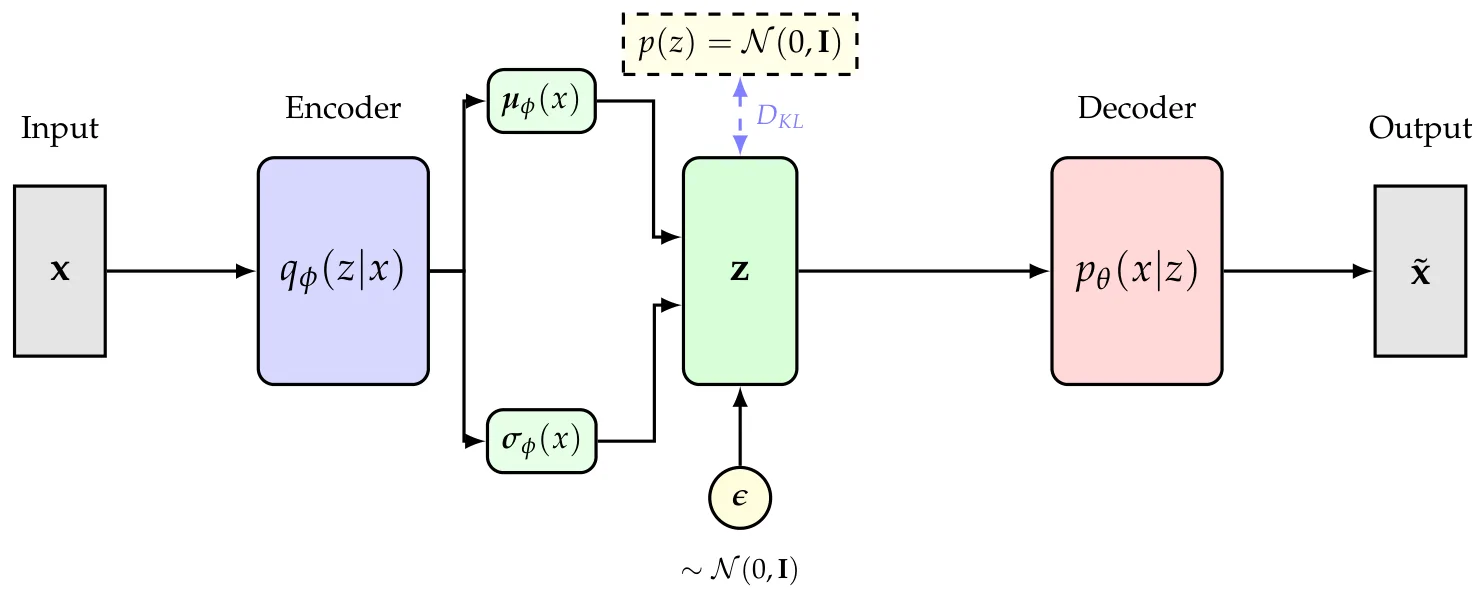
Variational Autoencoder architecture. The encoder maps input x to distribution parameters μ and σ. The latent variable z is sampled using the reparameterization trick with noise ϵ. The decoder reconstructs the input from z. The KL divergence regularizes the latent distribution towards the prior p(z).

**Figure 4:**
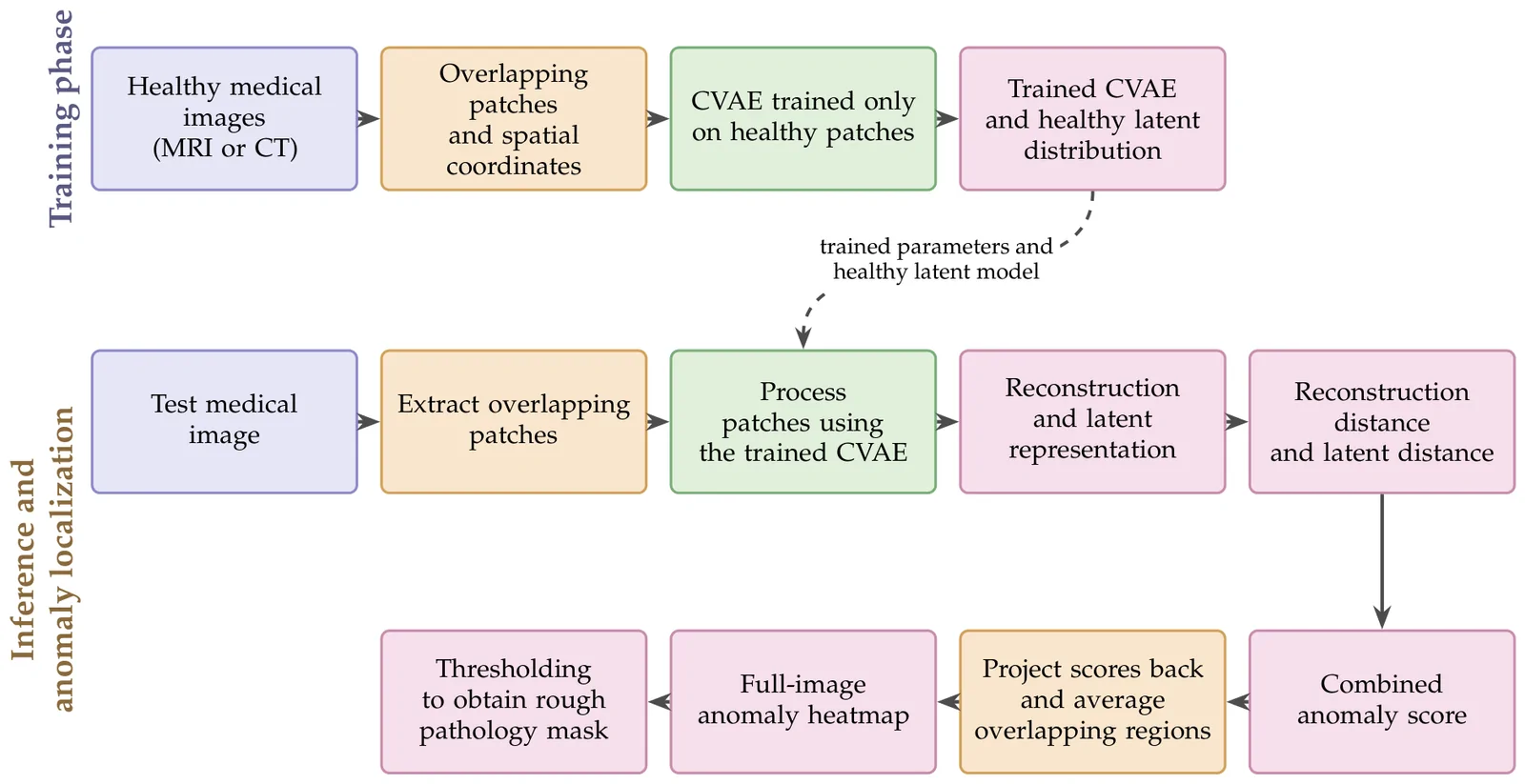
Workflow of the conditional variational autoencoder (CVAE)-based unsupervised pathology detection framework of Uzunova et al. [44].

**Figure 5:**
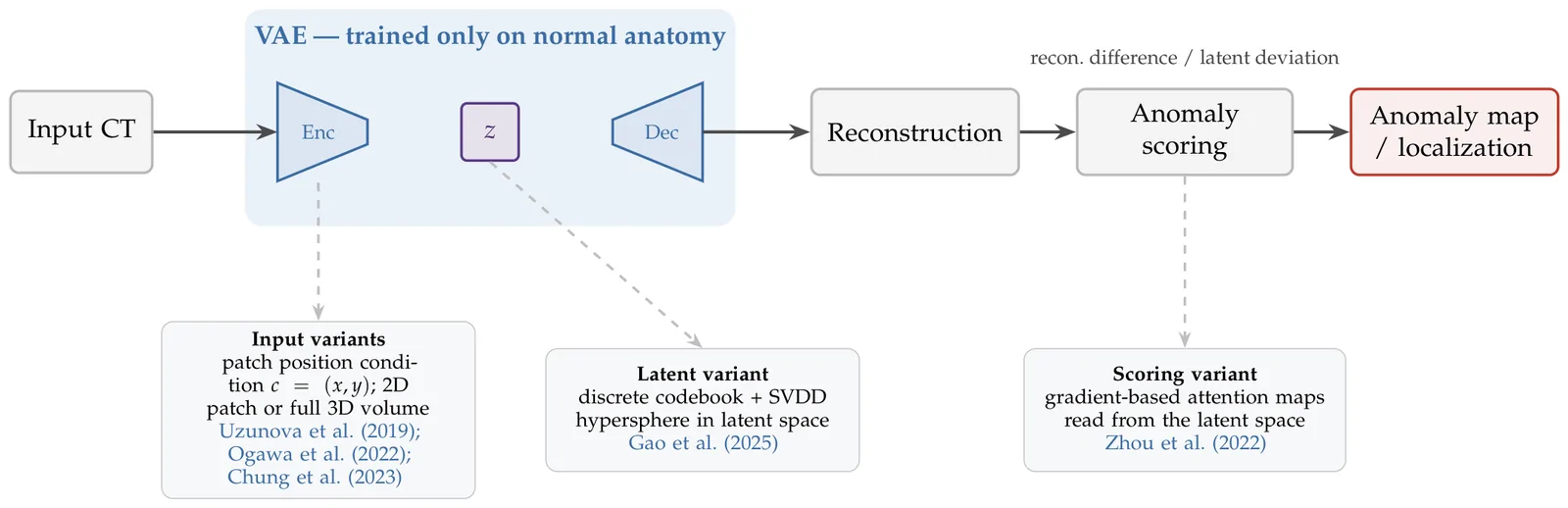
A unified view of VAE-based unsupervised anomaly localization in CT and related volumetric imaging. All methods share a single backbone (top): a VAE trained only on normal anatomy encodes the input, and the anomaly is flagged from the reconstruction difference or a latent-space deviation and then localized. The reviewed methods differ only at specific points of this backbone (bottom): the input may be position-conditioned and 2D or fully 3D [44,47,49]; the latent space may use a discrete codebook with an SVDD hypersphere [51]; and the anomaly readout may instead use gradient-based attention maps drawn from the latent space [50].

**Figure 6:**
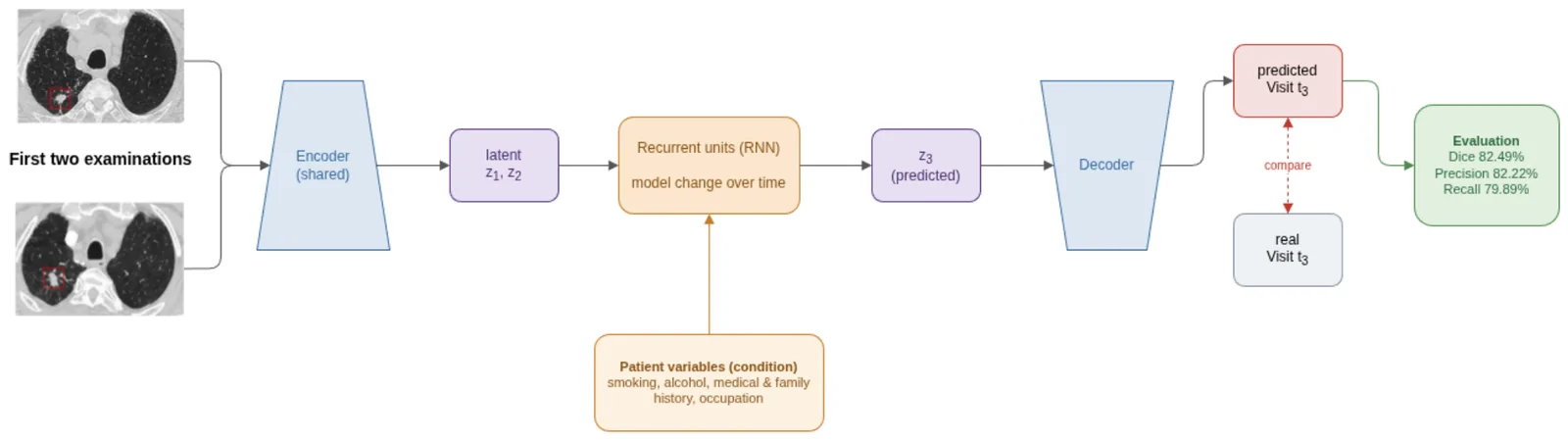
Workflow of the proposed CRVA model by Xiao et al. [55].

**Figure 7:**
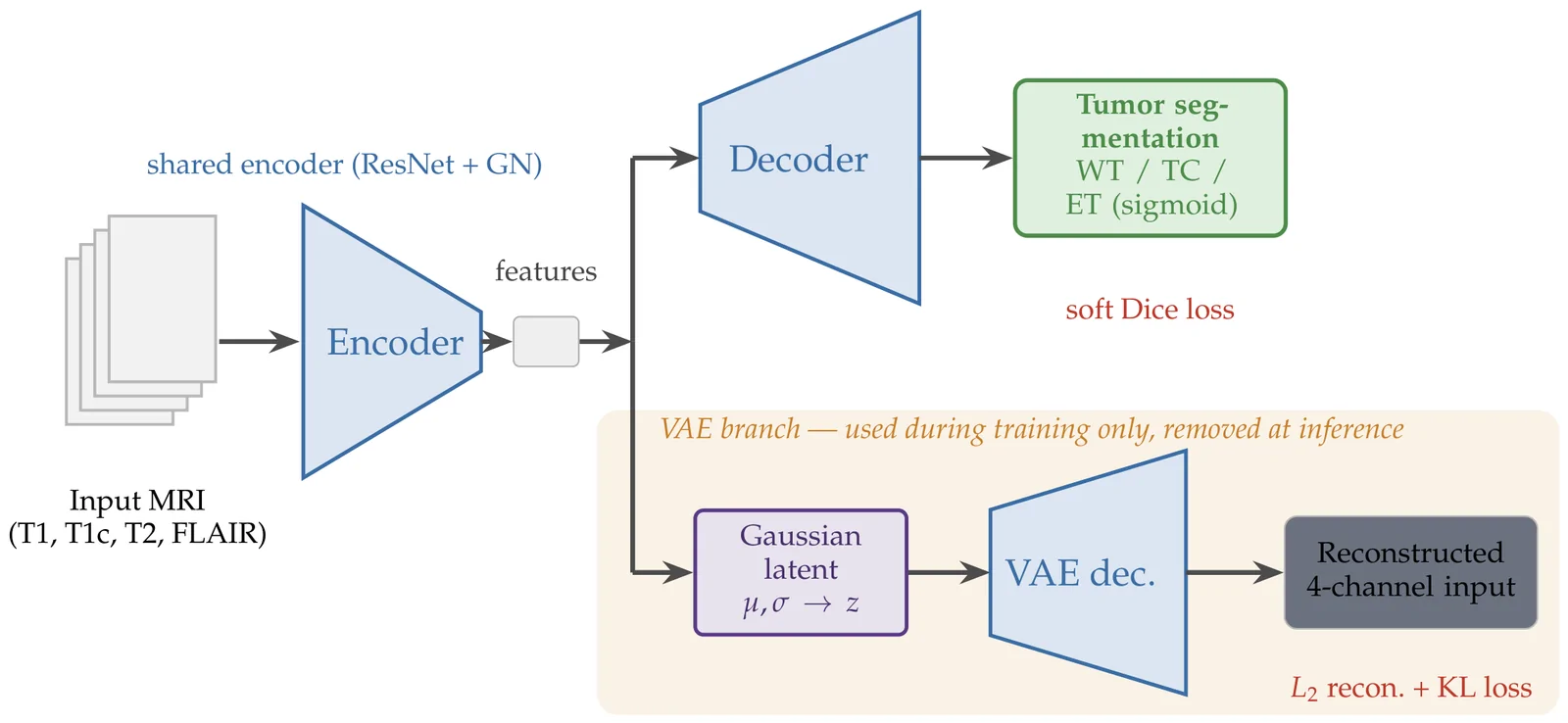
Architecture used in [65].

**Figure 8:**
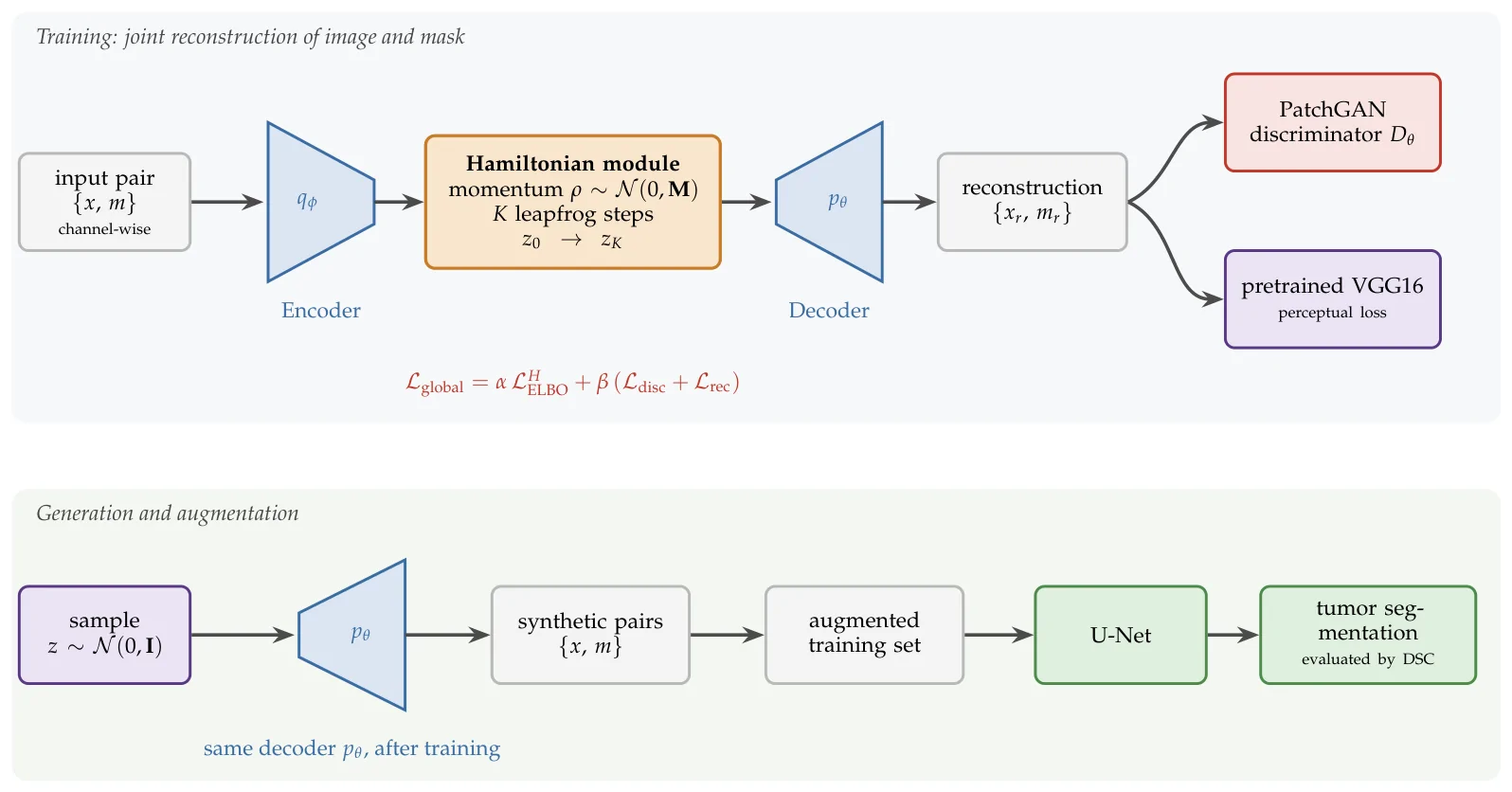
Architecture used in [71].

**Figure 9:**
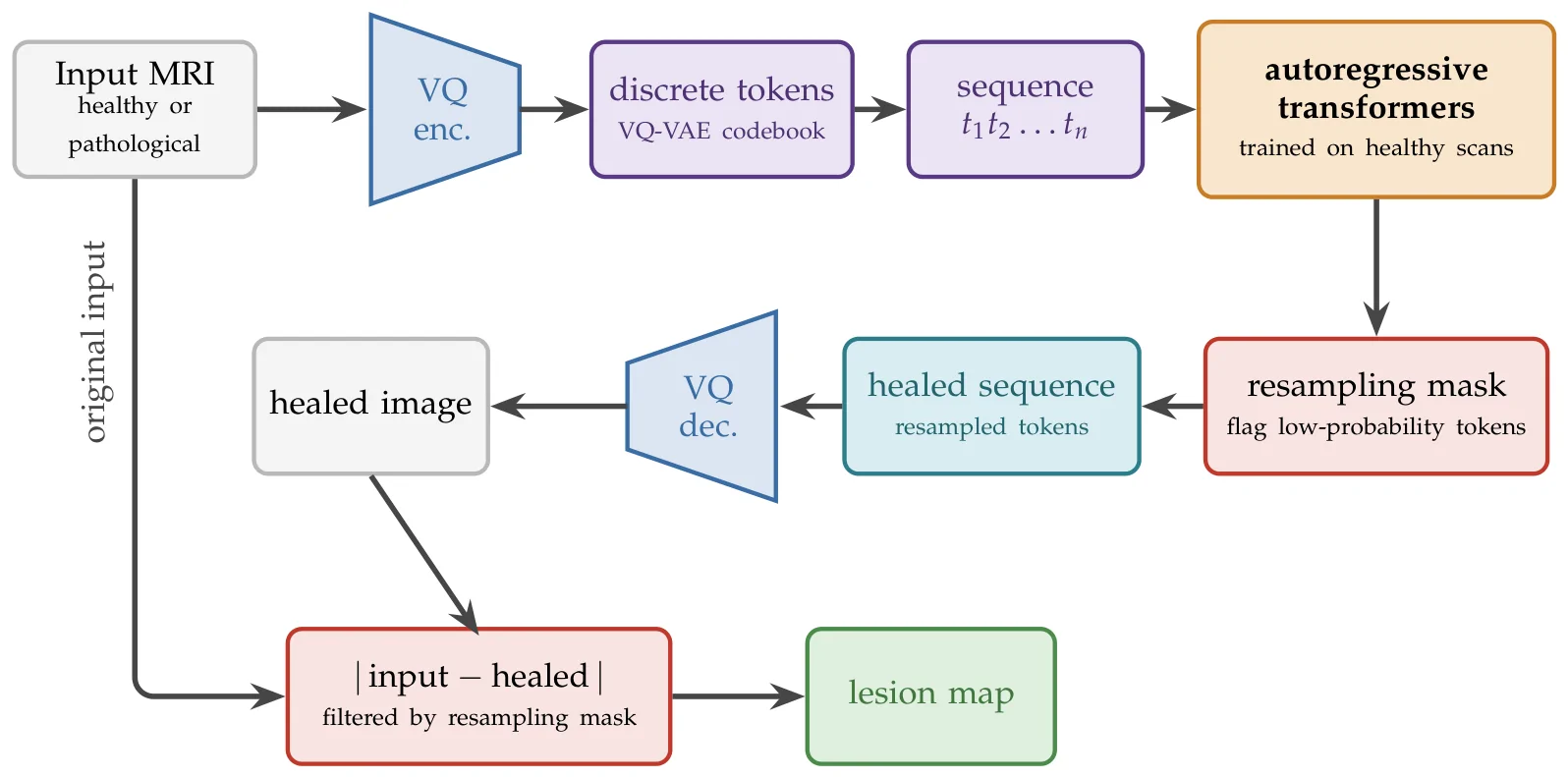
Restoration pipeline used in [87].

**Figure 10:**
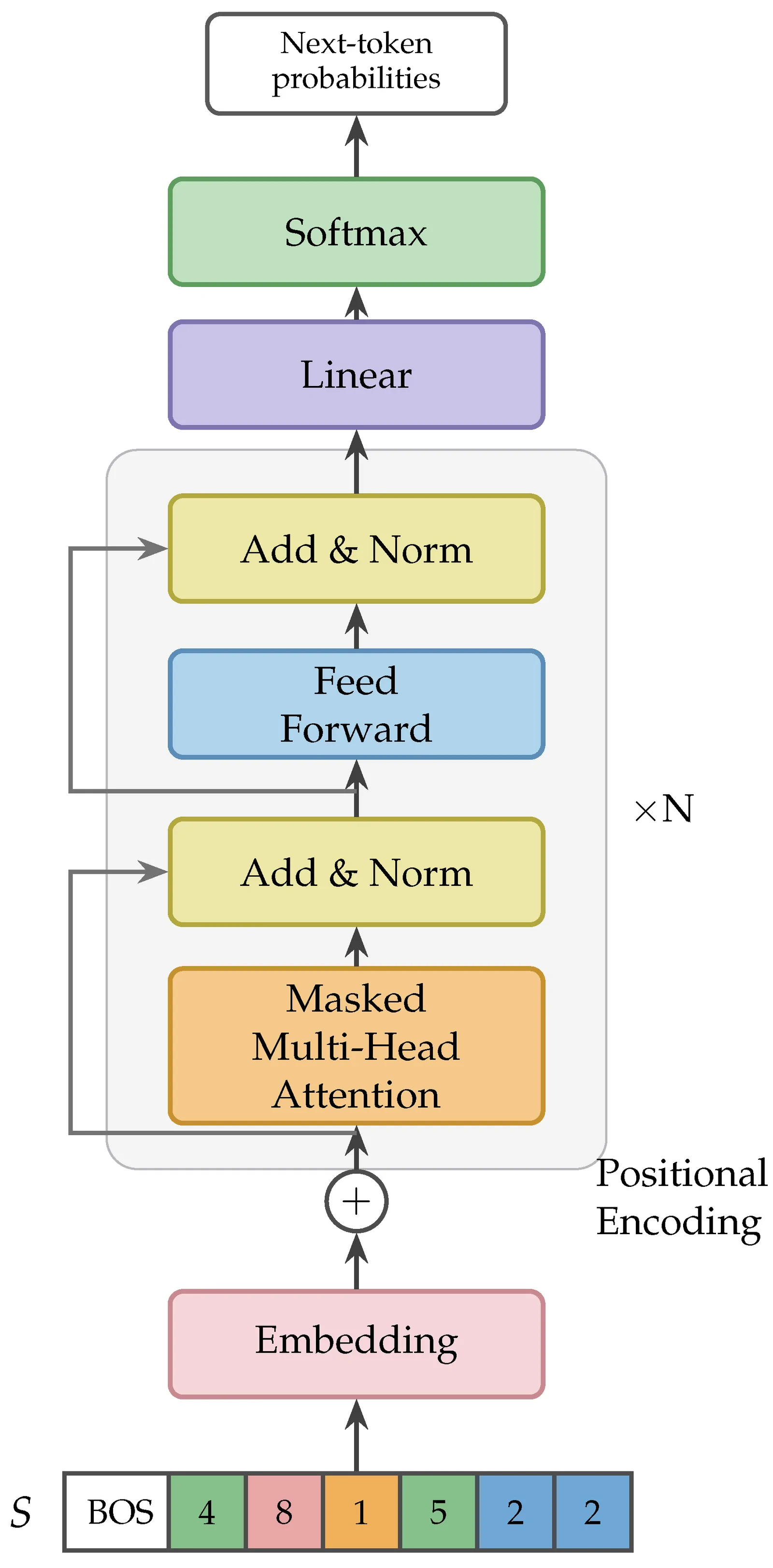
Autoregressive transformer used in [87].

**Figure 11:**
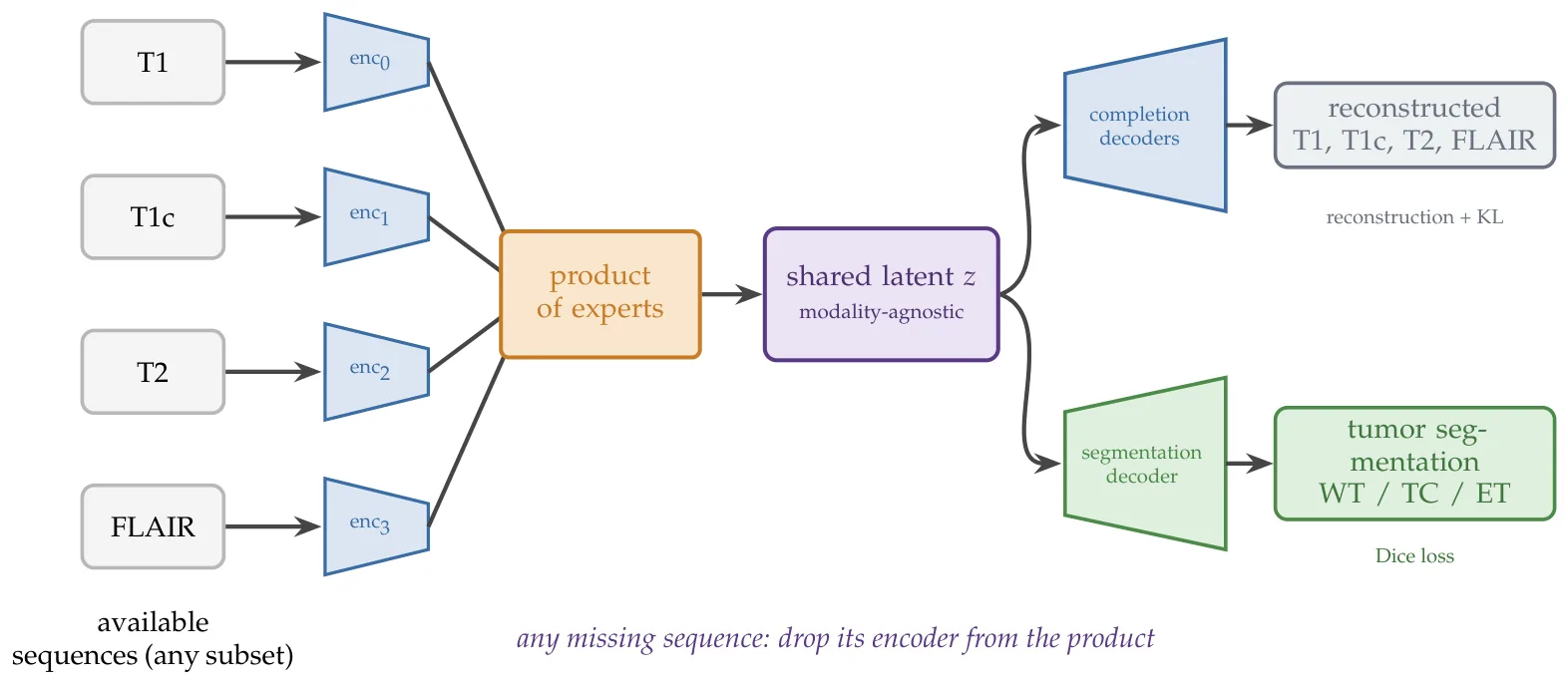
Architecture used in [116].

**Table 1:** Comparison of the present review with related reviews on generative models and medical image segmentation.

Review	Year	Main Scope	Modalities	Segmentation	VAEs	Missing Modalities
Yi et al. [4]	2019	GANs in medical imaging	Multiple	Included	No focus	No focus
Iqbal et al. [5]	2022	GANs for biomedical image segmentation	Multiple	Main	No focus	No focus
Zhou et al. [9]	2023	Brain tumor segmentation with missing MRI modalities	MRI	Main	Included	Main
Kebaili et al. [8]	2023	Generative models for medical image augmentation	Multiple	Downstream	Included	No focus
Kazerouni et al. [6]	2023	Diffusion models in medical imaging	Multiple	Included	No focus	No focus
Yeoh et al. [10]	2025	VAEs in structural medical imaging	Multiple	Included	Main	No focus
Yeoh et al. [11]	2026	Anatomy-specific VAE applications	Multiple	Included	Main	Included
Yaseen et al. [7]	2026	Diffusion models for medical image segmentation	Multiple	Main	No focus	No focus
**Present review**	**2026**	**Roles of VAEs in medical image segmentation**	**Multiple**	**Main**	**Main**	**Main**

*Main*: a central topic of the review; *Included*: discussed as one component of a broader review; *Downstream*: segmentation is mainly considered as an application of generated or augmented data; *No focus*: not specifically addressed.

**Table 2:** Documented database searches used for the review update.

Database	Search Date	Year Limit	Records Exported
PubMed	25 September 2026	2018–2026	80
IEEE Xplore	25 September 2026	2018–2026	387
Scopus	25 September 2026	2018–2026	370
**Total**			**837**

**Table 3:** Summary of VAE variants and their key characteristics.

Variant	Key Modification	Main Advantage
CVAE [15]	Conditioning on labels	Controlled generation
β-VAE [16]	Weighted KL term	Disentangled representations
VQ-VAE [17]	Discrete latent space	Discrete representation learning
VAE-GAN [19]	Feature-wise reconstruction	Sharper images
MVAE [22]	Product-of-experts posterior	Handles missing modalities
MMVAE [23]	Mixture-of-experts posterior	Robust cross-modal generation
MoPoE-VAE [24]	Mixture of products of experts	Consistent over modality subsets

**Table 4:** Summary of the VAE-based methods and related applications in radiographic imaging reviewed in this section.

Study	VAE Variant	Task	Dataset(s)	Key Results
*Latent representation integration and regularization (Role B)*				
Selvan et al. [25]	VAE + U-Net with a shared decoder	Lung segmentation under opacification	Shenzhen, Montgomery (train); COVID-19 CXR (test)	Dice 0.85, accuracy 0.88 (vs. U-Net Dice 0.82)
Cao and Zhao [28]	Fusion VAE at every encoder layer + three-terminal attention	Lung segmentation	Montgomery, Shenzhen (train); SNIH, JSRT (test)	Jaccard 0.882 (SNIH), 0.920 (JSRT)
Balaji [36]	3D Connected-UNets + VAE regularization	Breast tumor segmentation	INbreast, CBIS-DDSM, private mammography	TP 98%/TN 92% (INbreast); TP 95%/TN 90% (CBIS-DDSM)
*Related applications not assigned to a core taxonomy role*				
Ibrahim et al. [32]	VAEU-Net enhancement + ResUNet++ (two-stage)	Image enhancement followed by lung segmentation	JSRT, Montgomery	Dice 0.990 (JSRT), 0.985 (NLM-MC)
Rguibi et al. [33]	VAE-GAN (decoder as generator)	Chest X-ray synthesis	NIH ChestX-ray8	Inception score 1.84 (vs. 1.85 for real data)
Iglesias et al. [35]	MOC-VAE (reconstruction + classification + genetic saliency)	Explainable pathology localization	PadChest	Correct region in 80.56% of cases

**Table 5:** Summary of the VAE-based methods and related applications in CT imaging reviewed in this section. Datasets marked “in-house” are private clinical cohorts. Reported results are taken from each study’s own evaluation. Since the datasets, data splits, preprocessing, and the evaluation protocols are different across studies, the numerical values should not be directly compared across rows. Comparisons are most meaningful against the baselines reported within the same study.

Study	VAE Variant	Task	Dataset(s)	Key Results
*Variational representation integration and probabilistic segmentation (Role B)*				
Kohl et al. [39]	Conditional VAE + U-Net	Probabilistic lung-lesion segmentation	LIDC-IDRI [40]	Lower generalized energy distance than the compared baselines
Baumgartner et al. [41]	Hierarchical conditional VAE (PHiSeg)	Probabilistic segmentation and uncertainty modeling	LIDC-IDRI [40], in-house prostate MRI	GED 0.225 and NCC 0.845 on LIDC-IDRI (all experts; 100 samples)
Valiuddin et al. [42]	Probabilistic U-Net with latent-space regularization	Probabilistic segmentation/aleatoric uncertainty	LIDC-IDRI, QUBIQ	Up to 11% improvement in Hungarian-matched IoU
*Reconstruction-based anomaly localization (Role C)*				
Uzunova et al. [44]	Conditional VAE (patch, position-conditioned)	Pathology localization	BraTS, LPBA40, thoracic CT	Dice 0.55, AUC 0.95 (2D BraTS)
Ogawa et al. [47]	3D VAE	Inner-ear malformation	Temporal-bone CT (in-house, 6776)	AUC 0.99; SEN 99.1%, SPE 92.0%
Chung and Choi [49]	3D VAE	Extraocular-muscle enlargement	Orbital CT (in-house, 465)	AUROC 0.801; SEN 79.2%, SPE 72.7%
Gao et al. [51]	VQ-VAE + SVDD	Lung-lesion detection	Lung CT (1500)	AUC 0.76; SEN 0.68, SPE 0.73
Zhou et al. [50]	Context-encoding VAE + gradient attention	COVID-19 infection localization	MedSeg COVID-19 CT	Dice 0.559; SEN 66.7%, SPE 96.2%
*Related applications not assigned to a core taxonomy role*				
Li et al. [52]	3D β-VAE	Lesion representation learning	LIDC-IDRI, LNDb, Stanford radiogenomics	SSIM 0.774, PSNR 26.10, MSE 0.001
Xiao et al. [55]	Conditional recurrent VAE	Tumor-growth prediction	NLST + Shanxi hospital (880)	Dice 82.49%, Prec. 82.22%, Rec. 79.89%
Joseph et al. [57]	Feature-embedded VAE + perceptual loss	CBCT enhancement for downstream segmentation	CBCT → FBCT (in-house, IGRT)	High-quality FBCT synthesis
*Anatomical shape priors and segmentation quality control (Role E)*				
Sandfort et al. [58]	VAE trained on masks (shape prior)	Segmentation QC (liver/kidney/spleen)	Abdominal CT (12,495)	AUC 0.94/0.90/0.81; active learning 7.1% → 5.7%
Wang et al. [59]	VAE-based discriminator + CycleGAN	Annotation-efficient liver segmentation	CHAOS (CT/MRI)	Dice 0.933
Yao et al. [61]	VAE shape prior + teacher–student adaptation	Domain-adaptive pancreas segmentation	NIH, MSD, Synapse, in-house CT	Dice 0.757 MSD/0.787 Synapse

**Table 6:** Summary of the VAE-based methods for MRI segmentation reviewed in this section, grouped by role. Datasets marked “in-house” are private clinical cohorts. Reported results are taken from each study’s own evaluation. Since the datasets, data splits, preprocessing, and evaluation protocols differ across studies, the numerical values should not be directly compared across rows; comparisons are most meaningful against the baselines reported within the same study.

Study	VAE Variant	Task	Dataset(s)	Key Results
*Latent representation integration and regularization (Role B)*				
Myronenko [65]	3D encoder–decoder + VAE branch	Brain-tumor segmentation	BraTS 2018	Dice 0.766 ET/0.884 WT/0.815 TC (winner)
Lyu and Shu [67]	Two-stage cascade + VAE branch + attention gates	Brain-tumor segmentation	BraTS 2020	Dice 0.873 WT/0.836 TC/0.821 ET (test)
Tang et al. [68]	3D MultiResUNet + VAE regularization	Brain-tumor segmentation	BraTS 2020	Dice 0.703 ET/0.893 WT/0.790 TC
Pham et al. [69]	CNN + Transformer + VAE regularization	Brain/kidney tumor segmentation	BraTS 2021, KiTS19	Dice 85.48% ET/90.52% WT/92.60% TC
Habijan et al. [96]	3D FM-Pre-ResNet + VAE regularization	Whole-heart segmentation	MM-WHS (CT, MRI)	Dice 90.39% CT/89.50% MRI
Cui et al. [98]	VAMCEI + CycleGAN	Cross-modality cardiac MRI segmentation	MS-CMRSeg, MyoPS, MM-WHS	Mean Dice 85.96% (MS-CMRSeg)/81.20% (MyoPS)
Qiao et al. [103]	Contrastive margin-restrained VAE	Domain-adaptive segmentation	Cardiac MRI (cross-domain)	Improves target-domain Dice over whole-domain alignment
Liu et al. [75]	Variational inference + segmentation network	Probabilistic segmentation/inter-observer variability	QUBIQ brain-growth MRI	Continuous Dice 0.537
Zhou et al. [77]	Self-supervised VAE (EFS-MedSeg)	Few-shot multi-organ segmentation	OASIS, BCV, BCH, VerSe	Dice +1.4% OASIS/+9.1% BCV/+1.1% BCH
Yu et al. [76]	Segmentation Auto-Encoder with anatomical prior	Minimally supervised brain MRI segmentation	38 T1-weighted brain MRI scans	Dice 80.54%; HD95 2.68 mm
*Generative augmentation (Role A)*				
Kebaili et al. [71]	Discriminative Hamiltonian VAE	Data augmentation for tumor segmentation	BraTS 2021, HECKTOR 2022	FID 88.3; U-Net Dice 0.607 → 0.724 (BraTS)
Skandarani et al. [93]	VAE + SPADE-GAN	Synthetic cardiac MRI and mask generation	ACDC, Sunnybrook	Dice 0.776 → 0.874 (Sunnybrook)
*Reconstruction-based anomaly localization (Role C)*				
Baur et al. [80]	Comparative study (VAE variants)	Anomaly segmentation	MS and glioblastoma MRI	Normal/abnormal residual gap is the key indicator
Zimmerer et al. [81]	Context-encoding VAE (ceVAE)	Anomaly localization	BraTS 2017, ISLES 2015	AUROC 0.95 (BraTS), 0.89 (ISLES)
Chen and Konukoglu [82]	Constrained adversarial AE	Lesion detection	HCP + BraTS	AUC ≈ 0.90 → 0.92 with constraint
Bengs et al. [85]	3D VAE + spatial erasing	Anomaly segmentation	BraTS 2019	Dice 25.8% → 31.4% (2D → 3D+erasing)
Chatterjee et al. [86]	Compact ceVAE (StRegA)	Anomaly detection	BraTS (T2)	Dice 0.642 (real), 0.859 (synthetic)
Pinaya et al. [87]	VQ-VAE + autoregressive transformers	Anomaly detection/segmentation	UK Biobank, BraTS, MSLUB, WMH	Dice 0.617 BraTS/0.368 UKB/0.133 MSLUB
Wijanarko et al. [91]	Triplet VAE (Tri-VAE)	Brain-tumor detection	IXI (train), BraTS 2020	Dice 0.606, AUROC 0.968, AUPRC 0.462
*Anatomical shape priors and segmentation quality control (Role E)*				
Painchaud et al. [101]	Constrained/adversarial VAE (cVAE/aVAE)	Shape correction/QC	ACDC (MRI), CAMUS (echo)	Removes implausible shapes without lowering Dice
Biffi et al. [104]	Conditional VAE	3D cardiac segmentation reconstruction	UK Digital Heart Project (in-house)	Dice 87.92±0.15%; HD 3.99±0.03 mm

**Table 7:** Summary of the VAE-based methods for retinal vessel segmentation reviewed in this section, grouped by role. Reported results are taken from each study’s own evaluation. Since the datasets, data splits, preprocessing, and the evaluation protocols are different across studies, the numerical values should not be directly compared across rows. Comparisons are most meaningful against the baselines reported within the same study.

Study	VAE Variant	Task	Dataset(s)	Key Results
*Generative augmentation and pseudo-domain generation (Role A)*				
Hu et al. [105]	Full-resolution VAE (f-VAE) as pseudo-domains + Hessian vector field	Domain-generalized vessel segmentation	DRIVE, STARE, ARIA, HRF (fundus); OCTA-500, ROSE (OCTA)	Dice 0.706/0.573/0.641 on HRF subgroups; 0.779 OCTA-500; 0.728 ROSE (unseen targets)
Radha and Karuna [112]	Variational latent-space autoencoder + conditional GAN	Data generation for vessel segmentation	DRIVE, STARE, CHASE_DB	U-Net on synthetic data: Acc 0.996, AUC 0.990, IoU 0.983 (vs. 0.702 IoU on real)
*Latent representation integration and regularization (Role B)*				
Almarri et al. [114]	VAE + GAN (VGAN/GANVesselNet)	Supervised vessel segmentation	DRIVE, STARE	DRIVE Se/Sp/Acc 0.783/0.985/0.971; STARE 0.817/0.986/0.983

**Table 8:** Summary of the VAE-based methods for multimodal and missing-modality applications reviewed in this section, grouped according to their relationship with the taxonomy. For the segmentation studies, Dice scores are taken from each study’s own evaluation and use different BraTS editions and protocols, so they are not directly comparable across rows; each row is best read against the baselines reported in the same paper.

Study	VAE Variant	Task	Dataset(s)	Key Results
*Multimodal latent fusion and missing-modality segmentation (Role D)*				
U-HVED [116]	Hetero-modal VAE, product of experts, *n* encoders/n+1 decoders	Missing-modality tumor segmentation + completion	BraTS 2018	Avg. Dice 0.801 WT/0.640 TC/0.500 ET (vs. 0.507/0.287/0.274 for HeMIS)
RA-HVED [118]	Hetero-modal VAE + ROI-attentive skips + joint discriminator	Missing-modality segmentation	BraTS 2018, ISLES 2015	Avg. Dice 0.842 WT/0.756 TC/0.549 ET (vs. U-HVED 0.811/0.734/0.510)
XLSTM-HVED [119]	Hetero-modal VAE + Vision-xLSTM + self-attention encoder	Missing-modality segmentation + reconstruction	BraTS 2024	Avg. Dice 74.53 WT/58.19 TC/53.83 ET (vs. RA-HVED 68.82/47.82/39.77)
*Related application: missing-modality completion before segmentation*				
MGP-VAE [117]	Hetero-modal VAE with Gaussian-process prior	Missing-modality reconstruction followed by segmentation	BraTS 2019	Median Dice 0.835 WT/0.656 TC/0.507 ET (vs. 0.821/0.634/0.494 for HVED)
*Related cross-modality application: synthesis, not segmentation*				
MHVAE [120]	Multi-modal hierarchical VAE	MR–ultrasound synthesis	Brain MR, intra-op ultrasound	Shared hierarchical latent representation extends multimodal fusion beyond same-family MRI

**Table 9:** Public datasets used by the reviewed works, grouped by imaging area.

Dataset	Imaging Modality	Segmentation Target	Reference
**Chest X-ray**			
Montgomery (MC)	Chest radiograph	Lung fields	[26]
Shenzhen	Chest radiograph	Lung fields	[27]
JSRT	Chest radiograph	Lung fields, heart, nodules	[30]
SNIH	Chest radiograph (abnormal)	Lung fields	[29]
NIH ChestX-ray8	Chest radiograph	Thorax-disease labels (augmentation)	[34]
PadChest	Chest radiograph	Thorax findings (localization)	[121]
**Brain MRI**			
BraTS	Multi-sequence MRI (T1, T1c, T2, FLAIR)	Glioma sub-regions (WT/TC/ET)	[45]
ISLES 2015	Multi-sequence MRI	Ischemic stroke lesions	[83]
HCP	MRI	Healthy brain (normative)	[84]
UK Biobank	MRI	Brain (large-scale, normative)	[88]
MSLUB	MRI	Multiple sclerosis lesions	[89]
WMH	MRI (FLAIR, T1)	White-matter hyperintensities	[90]
IXI	MRI	Healthy brain (normative)	[92]
OASIS	MRI	Brain anatomical structures	[78]
QUBIQ	MRI/CT	Multi-rater anatomical segmentation	[43]
**Cardiac**			
ACDC	Cine MRI	Cardiac structures (LV, RV, myocardium)	[94]
CAMUS	Echocardiography (ultrasound)	Cardiac structures	[102]
Sunnybrook	Cine MRI	Left ventricle	[95]
MM-WHS	Cardiac CT/MRI	Whole-heart structures	[97]
MS-CMRSeg	Multi-sequence cardiac MRI	Cardiac structures	[99]
MyoPS	Multi-sequence cardiac MRI	Myocardium and myocardial pathology	[100]
**Abdominal and other CT**			
NIH Pancreas-CT	CT	Pancreas	[62]
MSD	CT/MRI	Multiple segmentation tasks; pancreas used here	[63]
Synapse/BCV	Abdominal CT	Multi-organ segmentation	[64]
CHAOS	Abdominal CT/MRI	Liver, kidneys, spleen	[60]
KiTS19	CT	Kidney and kidney tumor	[70]
VerSe	CT	Vertebrae	[79]
**Lung and chest CT**			
LIDC-IDRI	CT	Lung nodules	[40]
LNDb	CT	Lung nodules	[53]
NSCLC radiogenomics	CT	Lung tumors	[54]
NLST	CT	Lung (screening)	[56]
**PET/CT**			
HECKTOR	PET/CT	Head-and-neck tumors	[74]
**Mammography**			
INbreast	Digital mammography	Breast lesions/masses	[37]
CBIS-DDSM	Digital mammography	Breast lesions/masses	[38]
**Retinal**			
DRIVE	Color fundus	Retinal vessels	[106]
STARE	Color fundus	Retinal vessels	[107]
ARIA	Color fundus	Retinal vessels	[108]
HRF	Color fundus (high-resolution)	Retinal vessels	[109]
OCTA-500	OCT angiography	Retinal vessels	[110]
ROSE	OCT angiography	Retinal vessels	[111]
CHASE_DB	Color fundus	Retinal vessels	[113]

## Data Availability

No new data were created or analyzed in this study. Data sharing is not applicable to this article.

## Abbreviations

The following abbreviations are used in this manuscript:

Acc	Accuracy
ACDC	Automated Cardiac Diagnosis Challenge
ARIA	Automated Retinal Image Analysis
AUPRC	Area Under the Precision–Recall Curve
AUROC	Area Under the Receiver Operating Characteristic Curve
BraTS	Brain Tumor Segmentation (challenge)
CAMUS	Cardiac Acquisitions for Multi-structure Ultrasound Segmentation
ceVAE	Context-encoding Variational Autoencoder
CHASE_DB	Child Heart and Health Study in England (database)
CNN	Convolutional Neural Network
CT	Computed Tomography
CVAE	Conditional Variational Autoencoder
CXR	Chest X-ray
DRIVE	Digital Retinal Images for Vessel Extraction
DSC	Dice Similarity Coefficient
ELBO	Evidence Lower Bound
ET	Enhancing Tumor
FID	Fréchet Inception Distance
FLAIR	Fluid-Attenuated Inversion Recovery
GAN	Generative Adversarial Network
GN	Group Normalization
GP	Gaussian Process
HCP	Human Connectome Project
HD95	95th-percentile Hausdorff Distance
HECKTOR	Head and Neck Tumor (segmentation challenge)
HeMIS	Hetero-modal Image Segmentation
HRF	High-Resolution Fundus
HVED	Hetero-modal Variational Encoder–Decoder
IoU	Intersection over Union
ISLES	Ischemic Stroke Lesion Segmentation
JSRT	Japanese Society of Radiological Technology
KL	Kullback–Leibler (divergence)
LIDC-IDRI	Lung Image Database Consortium–Image Database Resource Initiative
MGP-VAE	Modality-completion Gaussian-Process-prior Variational Autoencoder
MHVAE	Multi-modal Hierarchical Variational Autoencoder
MMVAE	Mixture-of-experts Multimodal Variational Autoencoder
MoE	Mixture of Experts
MoPoE	Mixture of Products of Experts
MRI	Magnetic Resonance Imaging
MSLUB	Multiple Sclerosis dataset, University of Ljubljana
MVAE	Multimodal Variational Autoencoder
NLST	National Lung Screening Trial
OCTA	Optical Coherence Tomography Angiography
PoE	Product of Experts
PSNR	Peak Signal-to-Noise Ratio
RA-HVED	Region-of-interest Attentive Hetero-modal Variational Encoder–Decoder
ResNet	Residual Network
ROSE	Retinal OCT-Angiography Vessel Segmentation (dataset)
SAM	Segment Anything Model
Se	Sensitivity
Sp	Specificity
SSIM	Structural Similarity Index Measure
STARE	Structured Analysis of the Retina
TC	Tumor Core
U-HVED	U-Net Hetero-modal Variational Encoder–Decoder
VAE	Variational Autoencoder
VQ-VAE	Vector-Quantized Variational Autoencoder
WMH	White Matter Hyperintensities
WT	Whole Tumor
xLSTM	Extended Long Short-Term Memory
